# Human Milk Oligosaccharide Profiles over 12 Months of Lactation: The Ulm SPATZ Health Study

**DOI:** 10.3390/nu13061973

**Published:** 2021-06-08

**Authors:** Linda P. Siziba, Marko Mank, Bernd Stahl, John Gonsalves, Bernadet Blijenberg, Dietrich Rothenbacher, Jon Genuneit

**Affiliations:** 1 Pediatric Epidemiology, Department of Paediatrics, Medical Faculty, Leipzig University, 04103 Leipzig, Germany; Jon.Genuneit@medizin.uni-leipzig.de; 2 Danone Nutricia Research, 3584 CT Utrecht, The Netherlands; Marko.Mank@danone.com (M.M.); Bernd.Stahl@danone.com (B.S.); john.gonsalves@danone.com (J.G.); Bernadet.Blijenberg@danone.com (B.B.); 3 Department of Chemical Biology & Drug Discovery, Faculty of Science, Utrecht Institute for Pharmaceutical Sciences, Utrecht University, 3584 CG Utrecht, The Netherlands; 4 Institute of Epidemiology and Medical Biometry, Ulm University, 89075 Ulm, Germany; Dietrich.Rothenbacher@uni-ulm.de

**Keywords:** human milk oligosaccharides (HMOs), absolute quantitation, most abundant HMOs (trioses to hexaoses), targeted LC-MS/MS, stages of lactation, human milk groups, maternal secretor and Lewis (Le/Se) status

## Abstract

Human milk oligosaccharides (HMOs) have specific dose-dependent effects on child health outcomes. The HMO profile differs across mothers and is largely dependent on gene expression of specific transferase enzymes in the lactocytes. This study investigated the trajectories of absolute HMO concentrations at three time points during lactation, using a more accurate, robust, and extensively validated method for HMO quantification. We analyzed human milk sampled at 6 weeks (*n* = 682), 6 months (*n* = 448), and 12 months (*n* = 73) of lactation in a birth cohort study conducted in south Germany, using label-free targeted liquid chromatography mass spectrometry (LC-MS^2^). We assessed trajectories of HMO concentrations over time and used linear mixed models to explore the effect of secretor status and milk group on these trajectories. Generalized linear model-based analysis was used to examine associations between HMOs measured at 6 weeks of lactation and maternal characteristics. Results: Overall, 74%, 18%, 7%, and 1% of human milk samples were attributed to milk groups I, II, III, and IV, respectively. Most HMO concentrations declined over lactation, but some increased. Cross-sectionally, HMOs presented high variations within milk groups and secretor groups. The trajectories of HMO concentrations during lactation were largely attributed to the milk group and secretor status. None of the other maternal characteristics were associated with the HMO concentrations. The observed changes in the HMO concentrations at different time points during lactation and variations of HMOs between milk groups warrant further investigation of their potential impact on child health outcomes. These results will aid in the evaluation and determination of adequate nutrient intakes, as well as further (or future) investigation of the dose-dependent impact of these biological components on infant and child health outcomes.

## 1. Introduction

Human milk is considered the most suitable source of nutrition for infants. In addition to other biological components, human milk oligosaccharides (HMOs) are the third largest solid component of human milk and constitute approximately 20% of the total carbohydrate content [1]. More than 100 HMOs have been identified, but fewer than 20 typically account for more than 90% of the total HMO content in human milk [1]. Mature human milk typically contains 3–18 g/L of HMOs which are potentially indigestible for the infant [2,3]. Lower HMO concentrations have been reported in preterm milk (~15 g/L) compared to term milk (~17 g/L) and higher concentrations of HMOs have been reported in secretor milks (~18 g/L) [4]. In this case, secretor milk contains large amounts of specific HMO structures such as 2′-fucosyllactose (2′-FL) and lacto-N-fucopentaose I (LNFP I). The presence of such structures and others is therefore dependent on the expression of α1,2-fucosyltransferase Se- and Fuc-TIII (α1–3 fucosyltransferase) enzymes [5,6]. Based on this, human milk can be characterized into four milk groups [4,7,8,9,10], and it is only until recently that studies [11,12,13,14,15,16,17] have compared HMO concentrations between these four milk groups. Additionally, variations in HMO concentrations are attributable to general biological variability, lactation stage at sampling, geographic region [18,19], influences of other maternal factors, and differences in analytical methods [4]. Nonetheless, evidence suggests that HMOs function as prebiotics for the gut microbiota [20], possess antimicrobial activity [21], prevent pathogen binding and infections [22], modulate the immune system [23,24,25], and may also be necessary for brain [26,27] and cognitive development [28].

Although data on human milk are often used as a standard for establishing infant feeding regimens, few studies have reported HMOs as absolute concentrations in large sample sizes in real world observational cohorts. In addition, accurate, reliable, and robust methods for identifying and quantifying exact HMO concentrations are needed to identify specific concentrations of HMOs [4], as well as understand the exact concentrations at which they could impact infant health [29]. To our knowledge, only a few other studies [14,17,30,31,32] have investigated a variety of absolute individual HMO concentrations using current and recent liquid chromatography mass spectrometry (LC-MS) technologies. The methodology used in the current study was validated recently [33] and has overcome limitations from these previous studies, which include its sensitivity to measure more HMOs in a larger sample size. 

Moreover, most of the studies investigating HMO concentrations and their trajectories over time focused on the first few weeks of lactation. Some explored the composition of HMOs beyond one or two months [2,7,8,11,15,18,34,35,36,37] and we are aware of only two recent studies that investigated HMO concentrations up to 12 months [17] and 24 months [9] of lactation. Granted that individual HMOs have been shown to have specific effects on infant health and disease, there is a need for additional comprehensive research to elucidate the origin and implications of changes in HMO composition during lactation. 

In light of this, we measured absolute HMO concentrations in large and well-defined human milk sample sets in the Ulm SPATZ Health Study collected at 6 weeks, 6 months, and 12 months of lactation using a more accurate, robust, and extensively validated method, developed for HMO quantification. We further assessed the trajectories of HMOs over time, taking into account the four milk groups that were previously identified as well as the compositional nature of relative HMOs. Our study, therefore, aims to provide more information regarding longitudinal changes of HMOs during 12 months of lactation, adding to the existing literature on HMO variations during lactation.

## 2. Materials and Methods

### 2.1. Study Design and Population

Between April 2012 and May 2013, a total of 970 mothers and their 1006 new born infants were recruited from the general population during their hospital stay at the University Medical Centre Ulm in southern Germany for participation in the Ulm SPATZ Health Study [38]. If a mother lacked sufficient German language skills, had an outpatient childbirth, was under the age of 18, had a postpartum transfer of mother or her infant to intensive care unit, or had a still birth, she was excluded from participating in the study. Although participation was fully voluntary, all participants signed a written informed consent form prior to participation in the study. The ethics board of Ulm University granted ethical approval (No. 311/11).

### 2.2. Data Collection and Measurements

Demographic information (e.g., maternal age, education, pre-pregnancy body mass index (BMI), maternal smoking status in the 12 months prior to and during pregnancy), lifestyle, and birth-related data, such as child sex, mode of delivery, season in which the infant was born, and birthweight, were gathered using self-administered questionnaires. BMI was additionally computed as mass (kg)/height (m)^2^ using already-collected weight and height measurements. Electronic hospital charts and standard regular physical examinations were used to collect additional information. Human milk samples were collected at approximately 6 weeks (mean (SD), 5.9 (0.7) weeks, 6 months (25.8 (0.8) weeks), and 12 months (53.9 (4.3) weeks) post-delivery from willing lactating women who were still breastfeeding at the time at which samples were being collected. Instructions given to lactating mothers were to manually express or pump human milk between 9 a.m. and 12 p.m. Of note, this had to be done after breakfast and before lunch, but at least one hour after the infant′s last human milk feed. In some cases, whenever it was required, trained research nurses assisted mothers with human milk expression. Mothers were told to wash their hands and clean the breast prior to milk sampling, after which they were to manually express or pump human milk into two 10 mL collection tubes that were provided by the research study team. Mothers were further instructed to store the human milk samples in the fridge in their homes, until study nurses collected them either on the same day if milk was expressed between 9 a.m. and 12 p.m. (76.9%), or on the next day if milk was expressed in the evening (after 12 p.m., 9%; and before 9 a.m., 13.8%)). Upon collection, the samples were transported and delivered to the study center while refrigerated, after which they were further divided into aliquots within 48 h and kept frozen until further analysis. Additional study-related information was collected through interviews over the phone or using self-administered questionnaires delivered by mail at 6 weeks, 6 months, and 12 months post-delivery.

### 2.3. Analysis of HMOs

Human milk samples were kept frozen at −80 °C until HMOs were analyzed in 2019. Samples were transported to and received at Danone Nutricia Research on dry ice. The samples were defrosted overnight in a refrigerator (4–8 °C) and homogenized on a flatbed shaker for a duration of 30 min at room temperature. The samples were then gently inverted 10 times prior to aliquoting for subsequent analysis and kept at −80 °C. The identification and quantitation of individual native HMOs was accomplished by targeted liquid chromatography electrospray ionization tandem mass spectrometry (LC-ESI-MS^2^) in negative ion mode. This method allowed studying specific individual HMO structures which differed in the composition of monosaccharide, sequence, glycosidic linkages, or in the conformation of molecules. As a result, we could even identify isobaric HMO-isomers such as LNFP I, II, III, and V. The method outlined by Mank et al., 2019 [33] utilizing the concept of pre-defined precursor ions and collision induced (CID) diagnostic fragment ions for unambiguous identification and quantitation of lactose and 16 various (isomeric) HMOs was the basis for our LC-ESI-MS^2^ approach. For this study, the latter approach was adapted and improved in major aspects like choice of stationary phase and LC-gradient as described subsequently.

Clean up of human milk samples was achieved by delipidation and subsequent 3 kDa ultrafiltration (UF) in 96-well format. Aliquoted human milk samples (40 µL) were thawed for 15 min at room temperature and 200 µL of 80 mM ammonium formate (purity ≥ 99.0%, Sigma Aldrich, St. Louis, MO, United States of America (USA)) containing 0.12 g/L internal standard alpha-Arabinopentaose (purity ~90%, Megazyme, Bray, Ireland) was added [33]. The samples were briefly vortexed, and the lipids were separated by centrifugation at 20,000× *g* for the duration of 10 min at 4 °C. Then, 160 µL of the clear HMOs fraction was aspired carefully piercing through the lipid layer and by transferring the aspirate to a 350 µL 3 kDa UF 96-well plate (Omega membrane filter plate, Pall, New York, NY, USA) which was preflushed by centrifuging 200 µL of 67 mM ammonium formate in each well for one hour at 25 °C and 1500× *g*. A HMO filtrate was yielded by centrifugation at 1500× *g* for 2 h at 25 °C.

The filtrate was stored at −20 °C until it was required for further use. Prior to LC-ESI-MS^2^ analysis, the filtrate was thawed at RT, vortexed for one minute, 20 µL diluted 1:1 (*v*/*v*) with acetonitrile, and subjected to LC-ESI-MS^2^ analysis by injection of 5 µL sample. A 1200 series HPLC stack (Agilent, Waldbronn, Germany) which comprised solvent tray, degasser, binary pump, and auto sampler coupled to a 3200 Qtrap mass spectrometer (ABSciex, Framingham, MA, USA) was used to perform the LC-ESI-MS^2^ analysis. Oligosaccharides were then isolated using an Acquity UPLC BEH Amide Column (1.7 µM, 2.1 × 100 mm) (Waters, Milford, MA, USA) with a precolumn (1.7 µM 2.1 × 5 mm) (Waters, Milford, MA, USA). HMOs were separated using a 25 min water-acetonitrile gradient following a 4 min pre-equilibration step. Solvent A consisted of 10 mM ammonium formate and 0.05% ammonia in water and solvent B of 10 mM ammonium formate and 0.05% ammonia (Merck, Burlington, MA, USA) in 90% acetonitrile (VWR, Radnor, PA, USA). A 4 min pre-equilibration step was performed with a flowrate of 500 μL/min using 90% solvent B. The following gradient elution of HMOs was performed with a flowrate of 400 μL/min, starting with 90% solvent B for 0.5 min. Then, solvent B was decreased to 70% in 16 min. Subsequently, solvent B was further reduced to 50% in 0.5 min and after that was kept at 50% for 5 min. Then, B was increased to 90% in 0.1 min and was kept at 90% for 2.9 min. The column was constantly kept at 65 °C. HMO structures eluting from the column were injected into the mass spectrometer then analyzed qualitatively and quantitatively in negative ion mode using multiple reaction monitoring (MRM). For neutral HMOs up to hexaoses and acidic HMOs up to trioses, two distinct MRM transitions were monitored per HMO (Supplementary Table S1). The spray voltage and source temperature were −4500 V and 500 °C, respectively. Optimization of the declustering potential, entrance potential, exit potential, and collision energy were done for each compound measured. For optimizing the individual compounds 0.1 g/L in water was infused at 10 µL/min together with eluent (20% A + 80% B) at a flow rate of 200 µL/min and the [M-H]^−^ ion was selected. The optimal declustering MRM potential per compound was assessed by injecting a mixed standard and varying the declustering potential. Scheduled MRM was used with a target scan time of 2 sec and a 150 sec detection window. Calibration of the instrument with polypropylene glycol was done according to the manufacturer′s instructions. Absolute quantitation was achieved by an external calibration curve using standards obtained from Isosep (Tullinge, Sweden), Prozym (Ballerup, Denmark), Carbosynth (Berkshire, UK), Sigma Aldrich (MO, USA), and Elicityl (Crolles, France) of minimally 90% purity. Quantification of absolute HMO concentrations was done for lactose and 16 of the most abundant HMOs comprising: 2′-fucosyllactose (2′-FL); 3-fucosyllactose (3-FL); 3′-sialyllactose (3′-SL); 4′-galactooligosaccharide (4′-GL); 6′-galactooligosaccharide (6′-GL); 3,2′-difucosyllactose (DFL); 6′-sialyllactose (6′-SL); lacto-N-tetraose (LNT); lacto-N-neotetraose (LNnT); lacto-N-fucopentaose I (LNFP I); lacto-N-fucopentaose II (LNFP II); lacto-N-fucopentaose III (LNFP III); lacto-N-fucopentaose V (LNFP V); lacto-N-difusohexaose I (LNDFH I); and the sum of lacto-N-difusohexaose II and lacto-N-neodifucohexaose II (LNDFH II + LNnDFH II, standard containing both). 

Standards for quantitation (*n* = 8) were prepared in 20 mM ammonium formate, diluted 1:1 with acetonitrile, and injected together with the prepared samples in a batch. The calibration standards were run at the beginning, after 48 samples, and at the end of the batch. Two MRM transitions were monitored per compound, one served as a quantifier and one as a qualifier. The area ratio of the two transitions in the human milk samples had to be within 20% compared to the area ratio of the standards. This threshold was set to verify that no interference from other matrix compounds would affect the correct determination of HMO concentrations. The quality control (QC) sample was a colostrum milk sample attributed to milk group I and written informed consent form was obtained. A human milk sample batch consisted of 94 samples. The human milk-QC was prepared in duplicate together with the human milk study samples.

### 2.4. Method Validation

The abovementioned method was validated for repeatability, reproducibility, bias, batch stability, and linearity. The repeatability, reproducibility, and bias were determined in water on three different days at four different concentrations in triplicate to assess how calibration graphs were affected. The repeatability and bias in human milk were determined in three individual donors per milk group, fortified with HMOs, and performed in duplicate (*n* = 24). The reproducibility in human milk was calculated using the human milk-QC samples analyzed with the different batches. The linearity was investigated using prepared solutions (*n* = 8) with different known concentrations. The fit (linear or quadratic) and weighting (none, 1/× or 1/×2) were assessed for the best back-calculated concentration. To obtain a satisfying fit and weighting, a high and a low concentration range were determined for the oligosaccharides. If the compound concentration in the sample was less than the high concentration range, the compound concentration was calculated on the calibration graph for the low concentrations. To assess the stability of the LC-MS^2^ when analyzing a full batch of samples, the QC sample was prepared 96 times, pooled, and 40 µL was distributed in each of the 96 wells. The batch was injected and the RSD of the concentration per compound was calculated. The RSD was 4–8% except for 3′-SL and 6′-SL, which were 15% and 13%, respectively. To continuously monitor performance of the LC-ESI-MS^2^ analyses, a human milk-QC sample was introduced at the beginning and end of each human milk sample batch. Results of the validation are shown in Supplementary Table S1.

### 2.5. Secretor Phenotyping and Milk Group Assignment

The presence or absence of α1,2- and α1,4-fucosylated HMOs was used to classify human milk samples into different milk groups, as described in previous [2,5] and validated approaches [33]. Milk attributed to group I contains α1,2- and α1,4-fucosylated HMOs, for example 2′-FL and LNDFH I; milk that belongs to group II contains α1,4- fucosylated HMOs, which include LNDFH II, but lacks α1,2-fucosylated HMOs, like 2′-FL, LNFP I, and LNDFH I; group III milk contains α1,2-fucosylated HMOs, for instance 2′-FL and LNFP I, but with an absence of α1,4-fucosylated HMOs, like LNDFH I and II; and group IV, the least prevalent milk group, does not contain α1,2- nor α1,4- structures but comprises α1,3-fucosylated HMOs only, like 3-FL, which are present in milk attributed to either of the four groups since secretor or Lewis genes do not seem to affect their synthesis [5,11]. Briefly, in this study, lactating mothers whose milk contained both α1,2- and α1,4-fucosylated HMOs were attributed to group I. Women whose milk samples did not present LNFP I and LNDFH I (below the lower limit of quantification (<LLOQ)) were classified as group II. Women whose milk samples did not present LNFP II and LNDFH I (<LLOQ) were classified as group III. Those whose human milk samples did not present LNFP I, LNDFH I, and LNFP II (<LLOQ) were classified as type IV. For the categorization according to secretor phenotypes, milk groups I and III were grouped as secretors, while milk groups II and IV were grouped as non-secretors. HMO concentrations were presented as absolute values (g/L). The relative proportion of HMOs as percentages of total HMOs was then calculated from absolute values. The total HMO concentration was calculated by adding all the individual HMOs detected.

### 2.6. Statistical Analysis

Data for 4′-GL were excluded because >90% of values were found to be <LLOQ. For the remaining HMOs, all values <LLOQ were replaced by LLOQ/√ (2) and the values higher than the upper limit of quantification (>ULOQ) were extrapolated for 3-FL, 3′-SL, DFL, 6′-GL, 6′-SL, LNT, LNFP I, LNFP II, LNDFH I, and LNDFH II + LNnDFH II. The Kolmogorov–Smirnov test and visual inspection of histogram plots were used to ensure that the data had a normal distribution prior to analysis. Absolute HMO profiles were summarized using descriptive statistics and values are presented as mean (SD) as well as median (min, max). Relative and absolute HMO profiles were visualized and summarized using descriptive plots. Wilcoxon signed-rank test was used to compare HMO concentrations between 6 weeks, 6 months, and 12 months of lactation. The Blom′s normal ranks transformation [39] was applied to individual HMO concentrations before inclusion in the models. The Blom transformation allows an accurate approximation of the expected normal scores; therefore, results presented reflect the effect of a variable on a standardized one unit increase in HMOs. Associations between HMO concentrations measured at 6 weeks with secretor status, milk group, maternal BMI assessed prior to pregnancy, parity, mode of delivery, gestational age and maternal BMI at 6 weeks, infant sex, and exclusive breastfeeding were assessed using a general linear model. Associations were first assessed in univariate models then subsequently in a multivariate model. In addition, differences of HMO concentrations between secretor status and milk groups were assessed using a general linear model, with secretor and group I milk as reference groups, respectively. Changes in the total HMO concentration and lactose concentrations over lactation were investigated using a repeated measures general linear model. In subsequent group stratified analysis, we investigated the effect of maternal characteristics on the HMO trajectories over 6 weeks and 6 months, up to 12 months of lactation within milk groups I and II using linear mixed models. All models were first evaluated crude and subsequently adjusted for maternal age, maternal BMI, parity, exclusive breastfeeding, gestation period, and infant sex, unless otherwise stated. In additional analysis, the relative proportions of individual HMOs as percentages of total HMOs was calculated from the absolute values to determine overall HMO composition. Relative proportions of HMOs were transformed using centered log ratio (CLR) transformation to account for mutual dependency of compositional data. CLR is computed using the geometric mean of all constituents within a sample, in this case HMOs. Thus, CLR was computed as the natural log of the quotient of individual HMOs over the aforementioned geometric mean of all HMOs within one human milk sample. The effects of time, secretor status, and milk groups on HMO trajectories using both standardized and CLR-transformed HMOs were further determined using linear mixed effects models. Time, secretor status, and milk group were considered as fixed effects, and maternal IDs were entered in the models as random effects. Strengths and direction of associations are presented as Beta (β) estimates, with a positive coefficient indicating that as the value (Blom standardized) or constituent (CLR-transformed HMOs) of the independent variable increases, the mean of the dependent variable (i.e., HMO or lactose) also increases. The inverse is true for the negative estimates. The potential effects of multiple testing were controlled for using a Bonferroni-corrected level of statistical significance (α threshold = 0.05/16 = 0.0031). All statistical analyses were done using R (version 3.5.1; R Foundation for Statistical Computing) and SAS version 9.4 (The SAS Institute, Cary, NC, USA).

## 3. Results

Human milk samples were available for HMO analysis from 682, 448, and 73 lactating women at 6 weeks, 6 months, and 12 months, respectively (Table 1, Supplementary Tables S2 and S3). A total of 66 lactating mothers had HMO data available from samples collected at all three time points (6 weeks, 6 months, and 12 months). All women were aged 33.1, 33.5, and 34.3 years for the 6 weeks, 6 months, and 12 months samples, respectively. At 6 weeks, more than 70% of the lactating women were attributed to milk group I and 1% were attributed to milk group IV. In rare cases, i.e., 7 lactating women, they were initially attributed to milk group IV at 6 and 12 months. In the human milk samples of the aforementioned 7 lactating women, LNFPI was not present or <LLOQ in some cases, while other FUT2-related HMOs (2′-FL, DFL, LNDFH I) were found in partly quite high abundances. We then confirmed the milk groups by recognizing additional surrogate markers for active FUT2 and FUT3 (i.e., LNDFH I). As a result, these 7 human milk samples were re-assigned to milk group I.

### 3.1. HMO Concentrations at Different Timepoints of Lactation

#### 3.1.1. Absolute HMO Concentrations 

We assessed absolute HMO concentrations amongst lactating women who had HMO data available at both 6 weeks and 6 months (*n* = 422, Supplementary Table S4) and at all three time points (*n* = 66, Table 2). Following Bonferroni correction, most absolute HMO concentrations were significantly lower at 12 months of lactation (α threshold = 0.0031) than at earlier time points with the exception of 3-FL, DFL, 3′-SL, and LNDFH II + LNnDFH II, which were significantly higher (Table 2). The differences in HMO concentrations between 6 weeks and 6 months were the same when the sample size was restricted to the *n* = 422 mothers (Supplementary Table S4). As expected, individual and total HMO concentrations varied between the four milk groups at 6 weeks (Supplementary Table S5), 6 months (Supplementary Table S6), and 12 months (Supplementary Table S7). Overall, total combined HMOs (sum of 14 detected structures) in all human milk samples (mean ± SD; median (min, max)) were significantly (*p* < 0.0001) lower in group IV milk (3.02 ± 0.65; 2.88 (1.84, 4.19)) compared to group I (5.76 ± 1.22; 5.63 (2.49, 10.44)), group II (4.30 ± 1.07; 4.13 (1.66, 7.42)), and group III (6.44 ± 1.57; 6.52 (2.62, 10.67)).

#### 3.1.2. Relative Proportions (%) of HMO Concentrations

**Figure 1:**
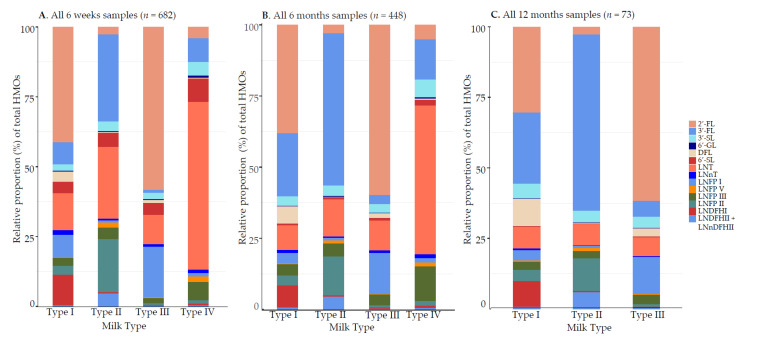
Relative proportion (%) of total human milk oligosaccharide (HMO) concentrations according to human milk group, in samples collected at 6 weeks, 6 months, and 12 months of lactation. Percentages of total HMOs were calculated for all women at (**A**) 6 weeks; (**B**) 6 months; and (**C**) 12 months according to the four different milk groups. 2′-FL, 2′-fucosyllactose; 3-FL, 3-fucosyllactose; 3′-SL, 3′-sialyllactose; 6′-GL, 6′-galactosyllactose; DFL, 3,2′-difucosyllactose; 6′-SL, 6′-sialyllactose; LNT, lacto-N-tetraose; LNnT, lacto-N-neotetraose; LNFP I, lacto-N-fucopentaose I; LNFP V, lacto-N-fucopentaose V; LNFP III, lacto-N-fucopentaose III; LNFP II, lacto-N-fucopentaose II; LNDFH I, lacto-N-difucohexaose I; LNDFH II, lacto-N-difucohexaose II; LNnDFH II, lacto-N-neodifucohexaose II.

**Figure 2:**
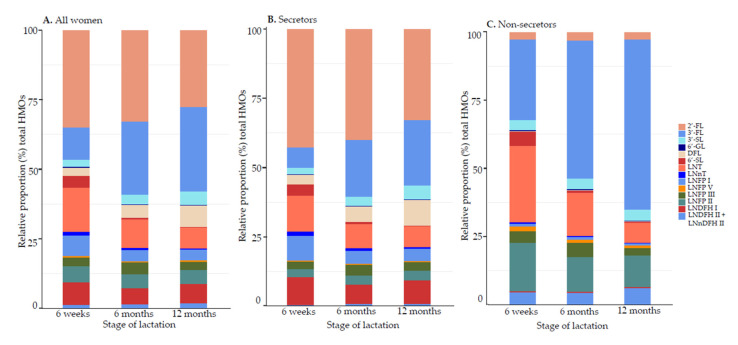
Relative proportion (%) of human milk oligosaccharides (HMOs) in human milk samples collected at 6 weeks, 6 months, and 12 months. Percentages of total HMOs were calculated for (**A**) all women regardless of milk group or secretor status; (**B**) secretor milks attributed to milk groups I and III; and (**C**) non-secretor milks attributed to milk groups II and IV. 2′-FL, 2′-fucosyllactose; 3-FL, 3-fucosyllactose; 3′-SL, 3′-sialyllactose; 6′-GL, 6′-galactosyllactose; DFL, 3,2′-difucosyllactose; 6′-SL; 6′-sialyllactose, LNT, lacto-N-tetraose; LNnT, lacto-N-neotetraose; LNFP I, lacto-N-fucopentaose I; LNFP V, lacto-N-fucopentaose V; LNFP III, lacto-N-fucopentaose III; LNFP II, lacto-N-fucopentaose II; LNDFH I, lacto-N-difucohexaose I; LNDFH II, lacto-N-difucohexaose II; LNnDFH II, lacto-N-neodifucohexaose II.

The relative proportion of 3-FL was more abundant in group II milk and LNT in group V milk (Figure 1, and Supplementary Tables S5–S7). When lactating mothers were stratified according to secretor phenotype, non-secretor mothers produced significantly lower concentrations of HMOs compared with their secretor counterparts (Figure 2, Supplementary Tables S8 and S9). In secretor milk, 2′-FL accounted for more than 40% of total detected HMOs. Within the secretor groups, total HMOs irrespective of time point were variable between lactating mothers in terms of HMO content (range among secretors, 2.62–12.60 g/L; non-secretors 1.66–8.64 g/L) as well as in relative composition (e.g., 2.71–77.30% 2′-FL among secretors; 0.89–65.20% LNT among non-secretors; Supplementary Tables S8 and S9). Most of the other HMOs also differed significantly by secretor status, except 6′-GL, 6′-SL, and LNFP III. 

#### 3.1.3. Trajectory of Lactose and HMOs during Lactation

**Figure 3:**
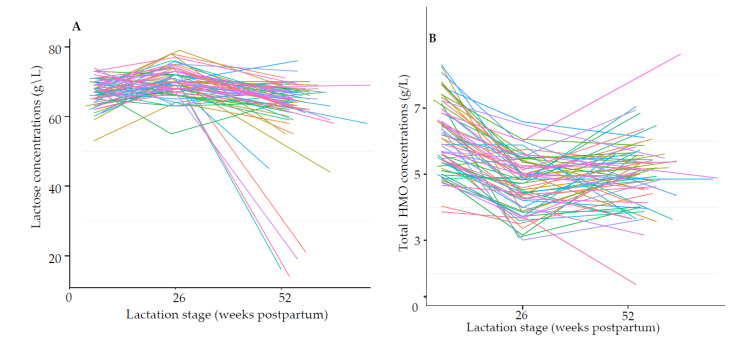
Multiple line plot of absolute (**A**) lactose and (**B**) total human milk oligosaccharide (HMO) concentrations (g/L) during lactation. Each line represents 1 sample among the lactating mothers with human milk samples collected at all three time points, 6 weeks, 6 months, and 12 months (*n* = 66). Lactation stage was determined by the real age of the infant at the stage of lactation at which their respective mothers provided a human milk sample.

A spaghetti plot showed a clear decline of lactose (Figure 3A) and total absolute HMO concentrations (Figure 3B) by 12 months of lactation. Subsequent analysis showed that lactose concentrations increased at 6 months (β = 0.6922, *p* < 0.0001) and decreased significantly at 12 months (β = −0.5602, *p* = 0.0002) compared to 6 weeks of lactation. These changes remained significant even after excluding the 6 mothers whose lactose concentrations were extremely low in comparison to the other values. Overall, total HMOs (all three time points combined) ranged from 1.66 to 12.65 g/L (mean 5.7 ± 1.35). Total HMO concentrations decreased significantly at 6 months (β = −1.3487, *p* < 0.0001) and 12 months (β = −1.0606, *p* < 0.0001) compared to 6 weeks of lactation, regardless of secretor status or milk group. These changes in lactose (Supplementary Figure S1A) and total HMO concentrations (Supplementary Figure S1B) were similar when the available data for 6 weeks and 6 months (*n* = 422) were considered.

#### 3.1.4. Effect of Time, Secretor Status, and Milk Group during Lactation 

We observed significant effects of time, secretor status, and milk groups on the trajectory of most individual HMOs at 6 months of lactation (*n* = 422, Supplementary Table S10a,b) and up to 12 months of lactation (*n* = 66, Supplementary Table S11a,b). These effects of time and milk group were statistically significant when evaluated within secretor, non-secretor, and the different milk groups at 6 months (Supplementary Table S12a,b) and up to 12 months of lactation (Supplementary Table S13a,b). 

### 3.2. Factors Associated with HMO Concentrations

#### 3.2.1. At 6 Weeks of Lactation

Following Bonferroni correction, secretor status was strongly associated with most individual HMO concentrations except 6′-SL (*p* = 0.4537), 6′-GL (*p* = 0.0523), and 3′-SL (*p* = 0.0478). Milk group was also associated with most individual HMOs except 6′-GL (*p* = 0.0953) and 6′-SL (*p* = 0.0353). Of the factors (i.e., pre-pregnancy BMI, parity, gestation period, delivery mode, exclusive breastfeeding, and infant sex), none of them were significantly associated with individual HMOs (α threshold = 0.0031, Supplementary Table S14). However, lactose was significantly higher in the milk for exclusively breastfed infants (β = 0.2788, *p* < 0.0001) and 6′-SL was higher (β = 0.1556, *p* = 0.0028) in the milk of overweight women at 6 weeks.

#### 3.2.2. Longitudinal Changes within Milk Groups I and II

We also investigated associations of individual HMOs with parity, delivery mode, gestation period, pre-pregnancy BMI, maternal BMI at 6 weeks, and exclusive breastfeeding in a stratified analysis (not shown) within milk groups I and II only. The small number of mothers whose milk was attributed to milk groups III and IV would limit conclusions. Of these factors, lactose was significantly lower in the milk of exclusively breastfed infants (β = −0.2824, *p* = 0.0007), amongst the mothers whose milk was attributed to milk group I (α threshold = 0.0031). Following correction for multiple testing, none of the factors examined were significantly associated with individual HMOs in the milk of lactating women attributed to group II (α threshold = 0.0031). We further investigated the effect of parity, gestation period, and maternal BMI on the changes in HMO concentrations at 6 months and up to 12 months of lactation within milk group I and II. Marginal associations of these factors with HMO trajectories over lactation were observed for some individual HMOs (Supplementary Tables S15 and S16), but none of these were statistically significant following adjustments for maternal age, pre-pregnancy BMI, infant sex, delivery mode, and multiple testing (α threshold = 0.0031).

## 4. Discussion

In the present study including lactating women from a birth cohort in Germany, we evaluated absolute HMO concentrations and their trajectories in human milk samples collected over 12 months of lactation, using a considerably more precise and comprehensively validated method developed for HMO quantification. Our results show that HMO concentrations are highly variable between mothers and change considerably over the course of lactation. The overall total content of HMOs decreased while some individual structures increased. The variations observed between mothers were strongly associated with milk group and secretor status. Milk group was also significantly associated with changes in HMOs within the secretor mothers. None of the other factors we examined (i.e., parity, gestation period, pre-pregnancy BMI, infant sex, mode of delivery, and exclusive breastfeeding) were significantly associated with at least one HMO at 6 weeks and their changes up to 6 months and 12 months of lactation.

The novelty of this study includes the quantification method (negative ion mode targeted MRM LC-ESI-MS^2^) used to determine absolute HMO concentrations in human milk sampled repeatedly across lactation in a large number of mothers. In comparison to other HMO-analytical approaches which only allow relative quantification, the method used in this study offered a higher efficiency of detecting HMOs. The enhanced selectivity of our method for highly similar HMO structures based on the concept of diagnostic tandem MS fragment ions allowed a precise identification and quantitation of even partly isomeric HMOs. This also facilitated assignment of individual human milk samples to one of the known four milk groups [33]. We measured lactose and 16 individual HMOs including the group isomers of LNDFH II and LNnDFH II which could not be separated but were measured together. The method employed in this study also allowed the separation of LNT and LNnT which were previously quantified together in another study [14] that also reported absolute concentrations quantified using LC-MS technology. Thus, the quantification method used in this study in particular eliminates the problem of co-elution of HMOs (of large concentrations) during quantification, the need to use uncommon HMO standards for identifying compounds during HMO quantification, as well as tedious derivatization of HMOs prior to analysis [33]. 

In addition, milk group I was the predominant milk group, and only 1% of the samples were attributed to milk group IV, which is known to be a rare milk group [5,14,16,40]. Although very rare [17,35,41], we identified seven lactating women in our study with peculiar HMO profiles. In principle, these lactating women were attributed to group IV milk at 6 and 12 months of lactation because of the complete absence or undetectable levels of LNFP I (<LLOQ). However, given the presence of 2′-FL in these human milk samples, we considered other FUT2-related HMOs such as (DFL, LNDFH I) which were present in partly quite high abundances to reliably confirm their milk group. The finding that LNFP I was absent or below LLOQ in a subgroup of group I human milk samples while other α1,2-fucosylated HMO-structures like 2′-FL, DFL, and LNDFH I were present is quite unusual.

A plausible explanation for this finding could be that a so far unknown α1,2-fucosyltransferase being active in parallel to the FUT2-encoded secretor enzyme produces LNFP I, which in turn is less active in the milks of these lactating women. Our findings may therefore indicate changes in the maternal genome with respect to the activation of FUT2 and possibly other FUTs that impact the synthesis of fucosylated HMOs in the mammary gland as lactation progresses. Although the reason for this phenomenon is unknown, our findings further confirm that milk group or secretor status may not be predicted based on a single HMO profiling-scheme within a human milk sample collected at one single time point of lactation [42]. Further insight into the genetic background of these lactating mothers or those with uncommon HMO profiles could aid researchers in their understanding of how HMO synthesis is regulated.

Consistent with other studies [2,7,8,9,11,15,16,18,34,36,37], our results show a considerable decline of the total content and most individual HMOs over lactation, irrespective of milk group or secretor status. However, 3-FL, DFL, and 3′-SL concentrations were consistently higher at 6 months and 12 months of lactation. On one hand, other studies have also reported constant [36], higher [8,9,37], and lower [2,11,15] concentrations of 3′-SL. On the other hand, the increase of DFL concentrations over lactation has only been reported in one recent study [9]. It is plausible that the discrepancies in these results are attributable to several factors which include but are not limited to genetic variants, geographic regions, ethnicity, and other maternal factors.

Nonetheless, 3′-SL is among some of the specific HMO structures that have been detected within the intestine as well as the systematic circulation of infants receiving human milk [43,44,45]. Despite the efforts towards providing evidence suggesting an important role of HMOs on immune cells, these previously proposed effects remain understudied. Furthermore, infants whose mother′s milk had low concentrations of α1,2-fucosylated HMOs were more likely to have *Campylobacter*, calicivirus, and moderate-to-severe diarrhea compared to their counterparts whose mother′s milk contained higher concentrations of α1,2-fucosylated HMOs [46]. Therefore, the changes that we observed in HMO concentrations over the course of lactation suggest that there is a need for these individual HMO structures during early infancy and perhaps later in childhood. Thus, an adequate intake should be ensured during this period of critical growth and development.

Furthermore, we primarily used the concentrations of 2′-FL, LNFP I, LNFP II, and LNDFH I as proxies for FUT2 and FUT3 activities. In this study, HMO concentrations were highly variable between lactating mothers. The presence and absence of the functional FUT2- and FUT3-encoded enzymes is known to greatly influence the HMO profile of lactating mothers [7,11,12,14,15,16,17,47]. Our results are in line with other studies [11,12,14,15,16] that also reported the presence and absence of some individual fucosylated HMOs in the different milk groups. However, Kunz et al. [16] reported LNT as a major component in milk group II and Gabrielli et al. [12] higher concentrations of LNnT than LNT in milk group I. This was not the case in our study. Nonetheless, the highest concentrations of these HMOs were observed at 6 weeks followed by a gradual decrease of most individual structures, while others increased by 12 months of lactation. We also investigated the effect of other maternal factors on the trajectories of HMOs within milk groups I and II only. However, in this study maternal characteristics did not contribute largely and significantly as milk group does to the variability of HMOs and their trajectories over lactation. 

Moreover, classifying the samples according to secretor and non-secretor status, we observed large differences in the total amount of HMOs and the trajectories of individual HMO concentrations. Other studies [7,8,9,12,37,48,49] have also shown higher 3-FL in non-secretor milks compared to their secretor counterparts. Similar differences were also observed in this study, confirming that the relative levels of the fucosylated oligosaccharides are a result of the competition between the enzymes for a limited substrate [15,17]. The differences between the groups were largely attributed to the presence and absence of α1,2-fucosylated HMOs such as 2′-FL, LNFP I, and LNDFH I in secretor and non-secretor milks. The considerable effect of milk group within secretor and variations within non-secretor mothers also confirm that there are indeed other factors that play a role in overall HMO concentrations in human milk [37,50]. It is important to note that at least one other study [9] investigated the trajectory of individual HMOs beyond 12 months of lactation. While these studies are similar, several other differences exist. The researchers in that study [9] reported that 2′-FL did not change over the course of 24 months, while we report a significant effect of time at 12 months of lactation. Secondly, we report absolute values, relative proportions of HMOs, and CLR-transformed HMOs, which accounts for the compositional nature of HMOs thereof. Of note, relative proportions of HMOs will differ depending on the number of individual HMO structures measured. Lastly, secretor status in that study was determined by the presence and near-absence of 2′-FL and LNFP I. Yet, we report both milk group and the importance of considering several other additional structures as proxies for FUT2 and FUT3 activity to determine secretor status. 

Nonetheless, certain HMO structures in secretor and non-secretor milks have been associated with health outcomes in infancy. Higher incidence of diarrhea was observed among infants whose mother′s milks had low concentrations of 2′-FL (non-secretor milk) compared to those who received human milk with higher amounts of 2′-FL (secretor milk) [46]. Moreover, higher concentrations of FUT2-dependent HMOs such as 2′-FL were associated with lower risk of allergy at age 2 and 5 in infants with high hereditary risk of allergy [51]. Therefore, early exposure of the infant to FUT2-dependent HMOs such as 2′-FL could be an important temporal window of opportunity to improve child health and possibly growth outcomes. Furthermore, human milk remains the ultimate choice of nutrition during early infancy. It is only recently that HMOs have begun being synthesized on an industrial scale, and some HMOs have been added to infant formula milk [52,53,54]. Yet, more research is still needed to determine whether HMO supplementation ultimately improves infant formula milk in a way that mimics the documented benefits of human milk [53]. Thus, the efficacy thereof as an alternative infant feeding regimen when human milk is insufficient or not available could be the focus of future perspectives for HMO-related research.

Although the evidence is conflicting, several maternal factors like parity, pre-pregnancy BMI, mode of delivery, exclusive breastfeeding, and gestation period have been associated with some individual HMOs [8,10,11,12,14,15,16,19,40,48]. In contrast to these studies, we found marginal associations with parity, pre-pregnancy BMI, exclusive breastfeeding, gestation period, and infant sex. The effect sizes of these associations were small and no associations were statistically significant following adjustment for respective maternal characteristics and correction for multiple testing. However, these observations could be an indication of the subtle changes that are associated with early post-partum physiology, which in turn for some HMOs, results in additional variations in the glycosylation pathways attributable to pre-pregnancy BMI, parity, and mode of delivery [15]. 

The strengths of this study include the presentation of absolute HMO concentrations across lactation and the use of a more accurate and extensively validated method with high specificity for HMO quantification. Furthermore, milk groups and grouping milks into secretor and non-secretor milk explains a large part of inter-individual variability in HMO concentrations. Thus, the confirmation of the four milk groups and assignment of lactating mothers to these milk groups based on a very high number of HM samples adds new information to the limited existing literature. However, we do not cover 100% of the abundance of HMOs by quantifying 16 individual HMOs. In addition, we cannot rule out the potential effect of long-term storage between time of sampling and analysis of HMOs on the composition of HMOs measured in the current study. 

Despite the reduced number of human milk samples available at all three time points, few studies have assessed the variations and changes in HMO concentrations up to [17] and beyond 12 months [9] lactation. Still, we provided a much larger sample size at 6 weeks and 6 months of lactation, and at 12 months in comparison to these two studies [9,17], respectively. Therefore, this study provides information regarding changes in HMO concentrations during a long-term period of lactation which could be further used to look at associations with child health outcomes. In addition, the study protocol for the collection of human milk samples was standardized in order to reduce the variability of HMOs and potential between feed variations of human milk components. However, due to the lack of dietary information, we could not investigate its impact on HMOs, if any. Future studies should investigate the potential impact of HMO trajectories over lactation on child health and growth outcomes. Further exploration of the synergistic mechanisms by which other human milk components potentially influence HMO concentrations may also contribute additional information in the relevance of research in human milk composition.

## 5. Conclusions

In conclusion, HMO concentrations vary considerably between mothers and may change over the course of lactation. These changes could be an indication of the temporal relevance of HMOs and their unique role later in infancy and childhood. Our findings warrant further exploration of their physiological relevance for the breastfed infant. Findings from this study also showed that the variations in HMO profiles that exist between mothers are largely attributed to milk group and secretor status, which are in turn dependent on the maternal genome and expression of FUT2- and FUT3-encoded fucosyltransferases in the lactocytes. However, FUT2 and FUT3 activity were determined using the concentrations of 2′-FL, LNFP I, LNFP II, and LNDFH I as proxies. Thus, in some rare instances, milk typing based on a specific scheme of HMOs needs confirmation by recognizing additional surrogate markers for FUT2 and FUT3 activity as outlined above. This phenomenon warrants further research into the genetic background of and HMO regulation by these mothers who exhibit such HMO profiles. Furthermore, in future studies, low abundant short chain HMOs like 3′-GL, but also more complex, long chain HMO-structures beyond the size of hexaoses should be addressed. To date, little is known about these human milk compounds and therefore more detailed investigation of their concentration across lactation is warranted. 

## Figures and Tables

**Table 1:** Characteristics of lactating mothers whose human milk samples were available for human milk oligosaccharide (HMO) analysis in the Ulm SPATZ Health Study.

Characteristics	All 6 Weeks Samples (*n* = 682)		All 6 Months Samples (*n* = 448)		All 12 Months Samples (*n* = 73)	
	*n*	% or Mean	*n*	% or Mean	*n*	% or Mean
Mother						
Age	681	33.1	447	33.5	72	34.3
Maternal body weight (6 weeks) (kg)	587	70.5	385	69.9	58	70.8
Maternal BMI (6 weeks) (kg)	662	24.7	438	24.8	72	25.1
Parity						
0 births	368	54	243	54	42	57.5
≥1 births	314	46	205	46	31	42.5
Milk group						
I	502	74	330	74	55	75
II	122	18	80	18	13	18
III	49	7	32	7	5	7
IV	9	1	6	1	0	0
Infant						
Female	339	50	216	48	29	40
Male	343	50	232	52	44	60
Gestation weeks	682	38.7	448	38.8	73	38.6
Birth weight (g)	682	3272	448	3282	73	3292
Delivery mode						
Vaginal spontaneous	432	63	294	65	48	66
Elective caesarean	80	12	52	11	8	11
Emergency caesarean	118	17	66	14	13	18
Vaginal assisted	52	8	36	8	4	5	

Sums (*n*) may not always add up to total because of missing values for certain items. Percentages exclude missing values. Milk groups were based on quantification and presence of certain human milk oligosaccharides.

**Table 2:** Absolute concentrations of human milk oligosaccharides (HMOs) measured in g/L at 6 weeks, 6 months, and 12 months of lactation in the Ulm SPATZ Health Study.

HMO	6 Weeks (*n* = 66)	6 Months (*n* = 66)	12 Months (*n* = 66)	*p* ^1^	*p* ^2^	*p* ^3^
2′-FL						
Mean (SD)	2.45 (1.32)	1.65 (1.08)	1.43 (0.85)	<0.001	0.305	<0.001
Median (min, max)	2.60 (0.13, 6.00)	1.65 (0.13, 4.30)	1.45 (0.13, 3.30)			
3-FL						
Mean (SD)	0.64 (0.57)	1.24 (0.74)	1.52 (1.26)	<0.001	0.446	<0.001
Median (min, max)	0.44 (0.04, 2.40)	1.05 (0.08, 3.10)	1.05 (0.12, 6.90)			
3′-SL						
Mean (SD)	0.12 (0.03)	0.14 (0.04)	0.25 (0.12)	0.470	<0.001	<0.001
Median (min, max)	0.13 (0.08, 0.24)	0.14 (0.07, 0.34)	0.22 (0.09, 0.58)			
6′-GL						
Mean (SD)	0.02 (0.01)	0.01 (0.00)	0.01 (0.01)	<0.001	0.188	<0.001
Median (min, max)	0.01 (0.01, 0.04)	0.01 (0.00, 0.02)	0.01 (0.00, 0.03)			
DFL						
Mean (SD)	0.18 (0.14)	0.20 (0.14)	0.40 (0.41)	0.198	0.010	<0.001
Median (min, max)	0.15 (0.01, 0.56)	0.20 (0.01, 0.51)	0.27 (0.01, 2.20)			
6′-SL						
Mean (SD)	0.24 (0.09)	0.04 (0.02)	0.01 (0.00)	<0.001	<0.001	<0.001
Median (min, max)	0.22 (0.08, 0.53)	0.03 (0.01, 0.12)	0.01 (0.01, 0.03)			
LNT						
Mean (SD)	0.86 (0.44)	0.44 (0.28)	0.36 (0.24)	<0.001	0.038	<0.001
Median (min, max)	0.75 (0.15, 1.90)	0.41 (0.10, 1.50)	0.32 (0.05, 1.20)			
LNnT						
Mean (SD)	0.08 (0.05)	0.05 (0.05)	0.02 (0.02)	<0.001	<0.001	<0.001
Median (min, max)	0.07 (0.01, 0.23)	0.03 (0.01, 0.23)	0.02 (0.01, 0.13)			
LNFP I						
Mean (SD)	0.43 (0.37)	0.19 (0.21)	0.20 (0.21)	<0.001	0.696	<0.001
Median (min, max)	0.31 (0.04, 1.60)	0.09 (0.04, 1.10)	0.12 (0.04, 1.00)			
LNFP V						
Mean (SD)	0.03 (0.03)	0.02 (0.02)	0.02 (0.02)	0.641	0.996	0.598
Median (min, max)	0.02 (0.01, 0.14)	0.02 (0.01, 0.10)	0.02 (0.01, 0.08)			
LNFP III						
Mean (SD)	0.17 (0.07)	0.19 (0.07)	0.14 (0.05)	0.089	<0.001	0.002
Median (min, max)	0.18 (0.05, 0.31)	0.18 (0.07, 0.36)	0.13 (0.04, 0.30)			
LNFP II						
Mean (SD)	0.30 (0.40)	0.24 (0.24)	0.24 (0.24)	0.567	0.800	0.376
Median (min, max)	0.13 (0.04, 1.70)	0.15 (0.04, 1.40)	0.17 (0.04, 1.20)			
LNDFH I						
Mean (SD)	0.50 (0.34)	0.27 (0.19)	0.36 (0.26)	<0.001	0.033	0.012
Median (min, max)	0.54 (0.02, 1.30)	0.27 (0.02, 0.70)	0.35 (0.02, 0.90)			
LNDFH II + LNnDFH II						
Mean (SD)	0.06 (0.12)	0.06 (0.08)	0.08 (0.11)	0.002	0.441	<0.001
Median (min, max)	0.01 (0.01, 0.70)	0.03 (0.01, 0.30)	0.03 (0.01, 0.46)			
Total HMOs						
Mean (SD)	6.09 (1.05)	4.74 (0.84)	5.03 (1.05)	<0.001	0.093	<0.001
Median (min, max)	5.91 (3.86, 8.32)	4.84 (3.01, 6.59)	5.07 (1.66, 8.64)			

*p* values derived from Wilcoxon signed-rank test comparing HMO concentrations between ^1^ 6 weeks and 6 months, ^2^ 6 months and 12 months, ^3^ 6 weeks and 12 months. Bonferroni-adjusted level of statistical significance is α = 0.05/16 = 0.0031. HMO, human milk oligosaccharides. 2′-FL, 2′-fucosyllactose; 3-FL, 3-fucosyllactose; 3′-SL, 3′-sialyllactose; 6′-GL, 6′-Galactosyllactose; DFL, 3,2′-difucosyllactose; 6′-SL, 6′-sialyllactose; LNT, lacto-N-tetraose; LNnT, lacto-N-neotetraose; LNFP I, lacto-N-fucopentaose I; LNFP V, lacto-N-fucopentaose V; LNFP III, lacto-N-fucopentaose III; LNFP II, lacto-N-fucopentaose II; LNDFH I, lacto-N-difucohexaose I; LNDFH II, lacto-N-difucohexaose II; LNnDFH II, lacto-N-neodifucohexaose II.

## Data Availability

Due to participant consent and data protection, we may not be able to share the raw data. However, the authors are open to sharing aggregate data (for instance, absolute concentrations that have been included as supplementary material).

## Supplementary Materials

The following are available online at https://www.mdpi.com/article/10.3390/nu13061973/s1, Table S1: Human milk oligosaccharide analysis method validation; Table S2: Absolute concentrations (g/L) of individual human milk oligosaccharides in all available human milk samples at 6 weeks, 6 months, and 12 months of lactation in the Ulm SPATZ Health Study; Table S3: Relative proportion (%) of human milk oligosaccharides (HMOs) in all available samples at each time point in the Ulm SPATZ Health study; Table S4: Absolute concentrations of individual human milk oligosaccharides restricted to mothers with samples collected at both 6 weeks and 6 months of lactation in the Ulm SPATZ Health Study; Table S5: Absolute concentrations of human milk oligosaccharides according to milk groups at 6 weeks; Table S6: Absolute concentrations of human milk oligosaccharides according to milk groups at 6 months; Table S7: Absolute concentrations of human milk oligosaccharides according to milk groups at 12 months; Table S8: Absolute concentrations of human milk oligosaccharides (HMOs) in human milk samples from the Ulm SPATZ Health Study by secretor status; Table S9: Relative proportion (%) of human milk oligosaccharides (HMOs) in human milk samples from the Ulm SPATZ Health study by secretor status; Figure S1: Split violin plots of the distribution of (a) lactose and (b) total human milk oligosaccharide concentrations (g/L) during the first 12 months of lactation regardless of milk type and secretor status; Table S10a: *p*-values from linear mixed effect models showing the effect of time, secretor status, and milk group on absolute human milk oligosaccharide concentrations over 6 months of lactation among the (*n* = 422) lactating women; Table S10b: *p*-values from linear mixed effect models showing the effect of time, secretor status, and milk group on centered log-ratio (CLR)-transformed human milk oligosaccharide concentrations over 6 months of lactation among the (*n* = 422) lactating women; Table S11a: *p*-values from linear mixed effect models showing the effect of time, secretor status, and milk group on absolute human milk oligosaccharide concentrations over 12 months of lactation among the (*n* = 66) lactating women; Table S11b: *p*-values from linear mixed effect models showing the effect of time, secretor status, and milk group on centered log-ratio (CLR)-transformed human milk oligosaccharide concentrations over 12 months of lactation among the (*n* = 66) lactating women; Table S12a: Effect of time and milk group within secretors, and effect of time within non-secretors, milk groups I, II, and III on absolute human milk oligosaccharide concentrations over 6 months of lactation among the (*n* = 422) lactating women; Table S12b: Effect of time and milk group within secretors, and effect of time within non-secretors, milk groups I, II, and III on centered log-ratio (CLR)-transformed human milk oligosaccharide concentrations over 6 months of lactation among the (*n* = 422) lactating women; Table S13a: Effect of time and milk group within secretors, and effect of time within non-secretors, milk groups I and II on absolute human milk oligosaccharide concentrations over 12 months of lactation among the (*n* = 66) lactating women; Table S13b: Effect of time and milk group within secretors, and effect of time within non-secretors, milk groups I and II on centered log-ratio (CLR)-transformed human milk oligosaccharide concentrations over 12 months of lactation among the (*n* = 66) lactating women; Table S14. Associations of secretor status, parity, exclusive breastfeeding, and infant sex with human milk oligosaccharide concentrations measured at 6 weeks in the Ulm SPATZ Health Study; Table S15. Unadjusted^1^ and adjusted^2^ effects of maternal characteristics on the trajectories of human milk oligosaccharides in milk groups I and II over 6 months of lactation in the Ulm SPATZ Health Study; Table S16. Influence of maternal characteristics on the trajectories of human milk oligosaccharides in milk groups I and II over 12 months of lactation in the Ulm SPATZ Health Study.
