# Supplementary material for: Human Milk Oligosaccharide Profiles over 12 Months of Lactation: The Ulm SPATZ Health Study
Source: Nutrients. 2021 Jun 8;13(6):1973. doi: 10.3390/nu13061973 (PMC8228739; doi:10.3390/nu13061973)
Supplement: Supplementary file 1 [file nutrients-13-01973-s001.zip › nutrients-1241188-supplementary.pdf]

Table S1. Human milk oligosaccaharide analysis method validation.

Compound<br>Retention time (min)	Quantifier<br>Q1/Q2 (m/z)<br>(DP) (CE)	Qualifier<br>Q1/Q2 (m/z)<br>(CE)	Low calibration (g/l)[a]<br>High calibration (g/l)[a]<br>Fit and weighting	Concentration added to water (g/l)<br>(*n* = 9)	Bias (%)<br>Repeatability (%)<br>Reproducibility (%)	Concentration added to milk (g/l)<br>(*n* = 24)	Bias (%)<br>Repeatability (%)	Reproducibility QC sample (%)<br>(*n* = 26)	Bias +/- combined deviation (%)
Lactose<br>4.95	341.1/179<br>(-40) (-10)	341.1/161<br>(-10)	2.5-25<br>25-100<br>Quadratic, none	-<br>-<br>-<br>80	-/-/-<br>-/-/-<br>-/-/-<br>-2/2/3	-	-/-	4	x ± 4
2'-Fucosyllactose<br>6.60	487.1/325<br>(-45) (-12)	487.1/205<br>(-24)	-<br>0.19-7.5<br>Quadratic, none	0.19<br>0.56<br>2.49<br>6.0	-15/5/5<br>-4/3/3<br>-3/3/4<br>-5/4/4	1.25	-4/7	6	-4 ± 9
3-Fucosyllactose<br>7.25	487.1/179<br>(-40) (-14)	487.1/89<br>(-34)	-<br>0.038-1.5<br>Quadratic, 1/x	0.038<br>0.11<br>0.50<br>1.2	-16/5/4<br>10/3/5<br>0/2/2<br>-3/3/4	0.25	-13/4	17	-13 ± 17
3'-Sialyllactose<br>8.50	632.2/290<br>(-60) (-35)	632.2/142<br>(-46)	0.013-0.062<br>0.030-0.50<br>Quadratic, 1/x	0.013<br>0.038<br>0.17<br>0.40	17/8/7<br>-1/5/7<br>-2/5/5<br>3/4/4	0.083	-40/11	12	-40 ± 16
4'-Galactooligosaccharide<br>8.20	503.1/101<br>(-30) (-35)	503.1/161<br>(-17)	0.0013-0.0062<br>0.0030-0.050<br>Quadratic, 1/x	0.0013<br>0.0038<br>0.017<br>0.040	7/9/15<br>-8/10/14<br>4/7/7<br>8/4/4	0.0083	-31/12	-	-31 ± 16[b]
6'-Galactooligosaccharide<br>8.65	503.1/179<br>(-40) (-22)	503.1/221<br>(-24)	0.0013-0.0062<br>0.0030-0.050<br>Quadratic, 1/x	0.0013<br>0.0038<br>0.017<br>0.040	4/7/8<br>-14/9/13<br>8/6/15<br>18/7/4	0.0083	-38/15	-	-38 ± 19[b]
3,2'-Difucosyllactose<br>9.15	633.2/325<br>(-45) (-27)	633.2/205<br>(-17)	0.019-0.094<br>0.045-0.75<br>Quadratic, 1/x	0.019<br>0.056<br>0.49<br>0.60	9/5/5<br>13/8/9<br>1/2/2<br>-4/3/2	0.13	-17/7	6	-17 ± 9
6'-Sialyllactose<br>9.50	632.2/290<br>(-60) (-34)	632.2/572<br>(-32)	0.013-0.062<br>0.030-0.50<br>Quadratic, 1/x.	0.013<br>0.038<br>0.17<br>0.40	18/4/4<br>-3/10/10<br>1/8/9<br>3/4/6	0.083	-41/20	8	-41 ± 22

Lacto-N-tetraose<br>10.80	706.2/202<br>(-35) (-28)	706.2/142<br>(-34)	0.068-0.062<br>0.17-2.7<br>Quadratic, 1/x	0.068<br>0.20<br>0.90<br>2.2	11/3/2<br>6/5/4<br>13/2/2<br>15/3/3	0.45	-20/18	17	-20 ± 25
Lacto-N-neotetraose<br>11.00	706.2/263<br>(-35) (-24)	706.2/281<br>(-22)	0.013-0.062<br>0.031-0.50<br>Quadratic, 1/x	0.013<br>0.038<br>0.17<br>0.40	8/3/5<br>-12/7/6<br>8/3/4<br>-1/2/3	0.083	-35/16	19	-35 ± 25
Lacto-N-fucopentaose I<br>12.85	852.5/325<br>(-45) (-30)	852.5/205<br>(-45)	0.050-0.25<br>0.13-2.0	0.05<br>0.15<br>0.67<br>1.6	4/2/3<br>-8/7/8<br>4/2/3<br>4/4/3	0.33	-56/15	8	-56 ± 17
Lacto-N-fucopentaose V<br>13.20	852.5/544<br>(-40) (-20)	852.5/382<br>(-28)	-<br>0.013-0.25<br>Quadratic, 1/x	0.013<br>0.038<br>0.17<br>-	8/7/7<br>14/4/7<br>4/2/6<br>-/-	0.083	-45/9	20	-45 ± 22
Lacto-N-fucopentaose III<br>13.55	852.5/364<br>(-40) (-26)	852.5/179<br>(-34)	0.025-0.13<br>0.060-1.0<br>Quadratic, 1/x	0.025<br>0.075<br>0.33	11/5/7<br>-7/6/7<br>2/3/4<br>-12/2/2	0.17	-34/7	13	-34 ± 15
Lacto-N-fucopentaose II<br>13.65	852.5/348<br>(-35) (-26)	852.5/288<br>(-34)	0.050-0.25<br>0.13-2.0<br>Quadratic, 1/x	0.05<br>0.15<br>0.67	4/6/7<br>-7/7/6<br>0/2/4<br>2/3/3	0.33	-51/10	21	-51 ± 23
Lacto-N-difucohexaose I<br>15.25	998.4/205<br>(-50) (-52)	998.4/836<br>(-24)	0.025-0.13<br>0.062-1.0<br>Quadratic, 1/x	0.025<br>0.075<br>0.33	3/4/6<br>-3/9/9<br>12/4/4<br>10/5/5	0.17	-68/5	11	-68 ± 12
Lacto-N-difucohexaose II +<br>Lacto-N-neodifucohexaose<br>II<br>15.25	998.4/690<br>(-50) (-24)	998.4/528<br>(-30)<br>998.4/364<br>(-30)	0.013-0.062<br>0.031-0.50<br>Quadratic, 1/x	0.013<br>0.038<br>0.17<br>0.40	-/13/11<br>-/6/6<br>-/7/7<br>-/5/6	0.083	-56/6	12	-56 ± 13

DP: declustering potential; CE: collision energy; Combined deviation $\sqrt{repeatability\ milk^2 + reproducibility\ QC^2}$; a: expressed as concentration in milk, absolute concentration 6 time lower; b: Concentration 4-GL <LLOQ and 6-GL >ULOQ in QC sample, used averaged reproducibility in water

Table S2. Absolute concentrations (g/l) of individual human milk oligosaccharides in all available human milk samples at 6 weeks, 6 months and 12 months of lactation in the Ulm SPATZ Health Study.

HMOs	6 weeks (*n*=682)	6 months (*n*=448)	12 months (*n*=73)
2'-FL			
Mean (SD)	2.31 (1.39)	1.68 (1.08)	1.36 (0.84)
Median [Min, Max]	2.50 [0.13, 6.60]	1.70 [0.13, 4.80]	1.40 [0.13, 3.30]
3'-FL			
Mean (SD)	0.65 (0.54)	1.21 (0.71)	1.57 (1.24)
Median [Min, Max]	0.47 [0.03, 3.00]	1.00 [0.05, 3.90]	1.10 [0.12, 6.9]
3'-SL			
Mean (SD)	0.15 (0.05)	0.16 (0.05)	0.24 (0.12)
Median [Min, Max]	0.15 [0.05, 0.54]	0.15 [0.05, 0.57]	0.21 [0.09, 0.58]
6'-GL			
Mean (SD)	0.02 (0.01)	0.01 (0.01)	0.01 (0.01)
Median [Min, Max]	0.02 [0.00, 0.15]	0.01 [0.01, 0.04]	0.01 [0.00, 0.03]
DFL			
Mean (SD)	0.19 (0.17)	0.24 (0.23)	0.38 (0.40)
Median [Min, Max]	0.17 [0.01, 1.80]	0.22 [0.01, 2.90]	0.26 [0.01, 2.20]
6'-SL			
Mean (SD)	0.26 (0.10)	0.04 (0.02)	0.02 (0.00)
Median [Min, Max]	0.24 [0.05, 0.730]	0.03 [0.01, 0.29]	0.01 [0.01, 0.03]
LNT			
Mean (SD)	0.94 (0.50)	0.47 (0.29)	0.36 (0.24)
Median [Min, Max]	0.84 [0.09, 3.10]	0.41 [0.05, 1.80]	0.32 [0.05, 1.20]
LNnT			
Mean (SD)	0.09 (0.06)	0.05 (0.0)	0.02 (0.02)
Median [Min, Max]	0.07 [0.01, 0.38]	0.03 [0.01, 0.24]	0.02 [0.01, 0.13]
LNFP I			
Mean (SD)	0.51 (0.50)	0.21 (0.25)	0.19 (0.20)
Median [Min, Max]	0.39 [0.04, 4.10]	0.12 [0.04, 1.90]	0.12 [0.04, 1.00]
LNFP V			
Mean (SD)	0.04 (0.04)	0.02(0.02)	0.022 (0.02)
Median [Min, Max]	0.02 [0.01, 0.24]	0.02 [0.01, 0.12]	0.02 [0.01, 0.08]

LNFP III			
Mean (SD)	0.18 (0.08)	0.20 (0.08)	0.14 (0.06)
Median [Min, Max]	0.18 [0.03, 0.56]	0.19 [0.03, 0.45]	0.14 [0.04, 0.30]
LNFP II			
Mean (SD)	0.33 (0.38)	0.24 (0.23)	0.25 (0.23)
Median [Min, Max]	0.17 [0.04, 2.50]	0.15 [0.04, 1.40]	0.18 [0.04, 1.20]
LNDFHI			
Mean (SD)	0.53 (0.39)	0.30 (0.22)	0.35 (0.25)
Median [Min, Max]	0.57 [0.02, 1.90]	0.31 [0.02, 0.99]	0.35 [0.02, 0.90]
LNDFHII + LNnDFHII			
Mean (SD)	0.06 (0.11)	0.06 (0.07)	0.08 (0.11)
Median [Min, Max]	0.02 [0.01, 0.73]	0.03 [0.01, 0.44]	0.03 [0.01, 0.46]
Total HMOs			
Mean (SD)	6.24 (1.33)	4.87 (0.89)	5.00 (1.01)
Median [Min, Max]	6.18 [2.41, 12.6]	4.85 [1.84, 8.35]	5.02 [1.66, 8.64]

HMO, human milk oligosaccharides. 2’-FL, 2’-fucosyllactose; 3-FL, 3-fucosyllactose; 3’-SL, 3’-sialyllactose; 6'-GL, 6’-Galactooligosaccharide; DFL, 3,2’-difucosyllactose; 6’-SL, 6’-sialyllactose; LNT, lacto-N-tetrose; LNnT, lacto-N-neotetraose; LNFP I, Lacto-N-Fucopentaose I; LNFP V, Lacto-N-Fucopentaose V; LNFP III, Lacto-N-Fucopentaose III; LNFP II, Lacto-N-Fucopentaose II; LNDFH I, Lacto-N-difucohexaose I; LNDFH II, Lacto-N-difucohexaose II; LNnDFH II, Lacto-N-neodifucohexaose II.

Table S3: Relative proportion (%) of human milk oligosaccharides (HMOs) all available samples at each time point in the Ulm SPATZ Health study

**HMOs**	**6 weeks (*n*=682)**	**6 months (*n*=449)**	**12 months (*n*=73)**
2'-FL			
Mean (SD)	35.1 (19.1)	33.0 (18.7)	27.7 (16.7)
Median [Min, Max]	39.0 [1.55, 74.2]	35.8 [2.16, 77.3]	29.8 [1.50, 71.4]
3'-FL			
Mean (SD)	11.6 (11.0)	26.3 (17.0)	30.5 (19.6)
Median [Min, Max]	7.68 [0.31, 53.5]	21.6 [0.85, 75.5]	23.9 [3.29, 81.8]
3'-SL			
Mean (SD)	2.56 (0.94)	3.39 (1.10)	4.85 (1.98)
Median [Min, Max]	2.36 [0.84, 8.32]	3.22 [0.91, 9.38]	4.13 [2.00, 10.2]
6'-GL			
Mean (SD)	0.29 (0.12)	0.20 (0.10)	0.20 (0.09)
Median [Min, Max]	0.26 [0.07, 2.45]	0.18 [0.03, 1.03]	0.18 [0.09, 0.57]
DFL			
Mean (SD)	2.84 (2.26)	4.61 (3.61)	7.63 (8.59)
Median [Min, Max]	2.70 [0.12, 18.3]	4.72 [0.17, 34.7]	5.76 [0.12, 56.5]
6'-SL			
Mean (SD)	4.27 (1.84)	0.73 (0.49)	0.23 (0.09)
Median [Min, Max]	3.90 [0.78, 16.1]	0.61 [0.15, 4.58]	0.22 [0.14, 0.60]
LNT			
Mean (SD)	15.9 (10.0)	10.1 (7.49)	7.48 (4.87)
Median [Min, Max]	13.7 [1.33, 65.2]	8.58 [0.81, 61.8]	7.12 [0.73, 25.1]
LNnT			
Mean (SD)	1.34 (0.94)	0.93 (0.82)	0.50 (0.46)
Median [Min, Max]	1.15 [0.12, 7.72]	0.65 [0.15, 5.17]	0.37 [0.12, 2.71]
LNFP I			
Mean (SD)	7.33 (5.93)	3.97 (4.16)	3.88 (4.00)
Median [Min, Max]	6.37 [0.48, 32.4]	2.47 [0.62, 26.6]	2.39 [0.46, 18.6]
LNFP V			
Mean (SD)	0.62 (0.68)	0.47 (0.38)	0.45 (0.36)
Median [Min, Max]	0.33 [0.09, 5.23]	0.31 [0.13, 2.16]	0.32 [0.14, 1.69]
LNFP III			

Mean (SD)	3.03 (1.43)	4.14 (1.79)	2.93 (1.30)
Median [Min, Max]	2.87 [0.34, 9.18]	3.88 [0.51, 20.2]	2.80 [0.54, 6.31]
LNFP II			
Mean (SD)	5.85 (6.83)	5.07 (4.78)	5.05 (4.36)
Median [Min, Max]	2.73 [0.32, 29.8]	3.20 [0.52, 23.3]	3.60 [0.73, 19.8]
LNDFHI			
Mean (SD)	8.10 (5.36)	5.82 (3.80)	6.99 (4.55)
Median [Min, Max]	9.24 [0.16, 22.5]	6.54 [0.26, 15.4]	7.97 [0.23, 15.3]
LNDFHII + LNnDFHII			
Mean (SD)	1.09 (1.84)	1.26 (1.58)	1.62 (2.21)
Median [Min, Max]	0.26 [0.08, 9.95]	0.59 [0.13, 7.75]	0.62 [0.14, 10.3]

HMO, human milk oligosaccharides. 2′-FL, 2′-fucosyllactose; 3-FL, 3-fucosyllactose; 3′-SL, 3′-sialyllactose; 6'-GL, 6′-Galactooligosaccharide; DFL, 3,2′-difucosyllactose; 6′-SL, 6′-sialyllactose; LNT, lacto-N-tetrose; LNnT, lacto-N-neotetraose; LNFP I, Lacto-N-Fucopentaose I; LNFP V, Lacto-N-Fucopentaose V; LNFP III, Lacto-N-Fucopentaose III; LNFP II, Lacto-N-Fucopentaose II; LNDFH I, Lacto-N-difucohexaose I; LNDFH II, Lacto-N-difucohexaose II; LNnDFH II, Lacto-N-neodifucohexaose II.

Table S4: Absolute concentrations of individual human milk oligosaccharides restricted to mothers with samples collected at both 6 weeks and 6 months of lactation in the Ulm SPATZ Health Study

HMOs	6 weeks (*n*=422)	6 months (*n*=422)	*p* value
Lactose			
Mean (SD)	66.7 (3.66)	70.5 (4.24)	< 0.001
Median [Min, Max]	67.0 [52.0, 77.0]	71.0 [46.0, 85.0]	
2'-FL			
Mean (SD)	2.35 (1.41)	1.69 (1.08)	< 0.001
Median [Min, Max]	2.50 [0.13, 6.60]	1.70 [0.13, 4.80]	
3'-FL			
Mean (SD)	0.62 (0.51)	1.20 (0.70)	< 0.001
Median [Min, Max]	0.45 [0.03, 2.60]	1.00 [0.05, 3.60]	
3'-SL			
Mean (SD)	0.15 (0.04)	0.16 (0.05)	0.003
Median [Min, Max]	0.15 [0.05, 0.37]	0.15 [0.05, 0.57]	
6'-GL			
Mean (SD)	0.02 (0.01)	0.01 (0.01)	< 0.001
Median [Min, Max]	0.02 [0.01, 0.15]	0.01 [0.00, 0.04]	
DFL			
Mean (SD)	0.18 (0.16)	0.24 (0.23)	< 0.001
Median [Min, Max]	0.16 [0.01, 1.80]	0.22 [0.01, 2.90]	
6'-SL			
Mean (SD)	0.26 (0.10)	0.04 (0.02)	< 0.001
Median [Min, Max]	0.24 [0.05, 0.73]	0.03 [0.01, 0.12]	
LNT			
Mean (SD)	0.93 (0.51)	0.47 (0.29)	< 0.001
Median [Min, Max]	0.85 [0.09, 2.90]	0.41 [0.05, 1.80]	
LNnT			
Mean (SD)	0.086 (0.06)	0.05 (0.04)	< 0.001
Median [Min, Max]	0.0750 [0.0100, 0.340]	0.03 [0.01, 0.24]	
LNFP I			
Mean (SD)	0.50 (0.46)	0.21 (0.25)	< 0.001
Median [Min, Max]	0.38 [0.04, 3.00]	0.12 [0.04, 1.90]	

LNFP V			
Mean (SD)	0.03 (0.03)	0.02 (0.02)	< 0.001
Median [Min, Max]	0.02 [0.01, 0.17]	0.02 [0.01, 0.12]	
LNFP III			
Mean (SD)	0.17 (0.07)	0.19 (0.07)	< 0.001
Median [Min, Max]	0.17 [0.03, 0.48]	0.20 [0.03, 0.43]	
LNFP II			
Mean (SD)	0.32 (0.38)	0.23 (0.23)	0.189
Median [Min, Max]	0.16 [0.04, 2.00]	0.1503 [0.04, 1.40]	
LNDFHI			
Mean (SD)	0.52 (0.36)	0.30 (0.22)	< 0.001
Median [Min, Max]	0.57 [0.02, 1.90]	0.31 [0.02, 0.99]	
LNDFHII + LNnDFHII			
Mean (SD)	0.06 (0.10)	0.06 (0.07)	< 0.001
Median [Min, Max]	0.01 [0.01, 0.70]	0.03 [0.01, 0.44]	
Total HMOs			
Mean (SD)	6.18 (1.26)	4.86 (0.90)	< 0.001
Median [Min, Max]	6.17 [2.41, 11.6]	4.85 [1.84, 8.35]	

p values derived from Wilcoxon signed-rank test comparing HMOs concentrations between 6 weeks and 6 months. Bonferroni-adjusted level of statistical significance is α=0.05/16=0.0031. HMO, human milk oligosaccharides. 2′-FL, 2′-fucosyllactose; 3-FL, 3-fucosyllactose; 3′-SL, 3′-sialyllactose; 6'-GL, 6′-Galactooligosaccharide; DFL, 3,2′-difucosyllactose; 6′-SL, 6′-sialyllactose; LNT, lacto-N-tetrose; LNnT, lacto-N-neotetraose; LNFP I, Lacto-N-Fucopentaose I; LNFP V, Lacto-N-Fucopentaose V; LNFP III, Lacto-N-Fucopentaose III; LNFP II, Lacto-N-Fucopentaose II; LNDFH I, Lacto-N-difucohexaose I; LNDFH II, Lacto-N-difucohexaose II; LNnDFH II, Lacto-N-neodifucohexaose II.

Table S5: Absolute concentrations of human milk oligosaccharides according to milk groups at 6 weeks

HMOs	Group I (*n*=502)	Group II (*n*=122)	Group III (*n*=49)	Group IV (*n*=9)
Lactose				
Mean (SD)	66.3 (3.73)	67.2 (3.78)	66.4 (4.38)	66.9 (4.01)
Median [Min, Max]	67.0 [52.0, 77.0]	67.0 [56.0, 79.0]	67.0 [56.0, 74.0]	68.0 [57.0, 70.0]
2'-FL				
Mean (SD)	2.68 (0.88)	0.13 (0)	4.27 (1.04)	0.13 (0)
Median [Min, Max]	2.60 [0.13, 6.30]	0.13 [0.13, 0.13]	4.30 [1.00, 6.60]	0.13 [0.13, 0.13]
3'-FL				
Mean (SD)	0.48 (0.26)	1.57 (0.48)	0.08 (0.05)	0.30 (0.15)
Median [Min, Max]	0.425 [0.0900, 2.40]	1.60 [0.63, 3.00]	0.06 [0.03, 0.21]	0.25 [0.08, 0.58]
3'-SL				
Mean (SD)	0.15 (0.04)	0.18 (0.05)	0.16 (0.04)	0.16 (0.04)
Median [Min, Max]	0.14 [0.05, 0.54]	0.16 [0.07, 0.32]	0.15 [0.09, 0.34]	0.16 [0.08, 0.22]
6'-GL				
Mean (SD)	0.02 (0.01)	0.02 (0.01)	0.02 (0.01)	0.02 (0.01)
Median [Min, Max]	0.02 [0.00, 0.09]	0.02 [0.01, 0.15]	0.02 [0.01, 0.04]	0.02 [0.01, 0.04]
DFL				
Mean (SD)	0.24 (0.17)	0.01 (0)	0.10 (0.12)	0.01 (0)
Median [Min, Max]	0.21 [0.02, 1.80]	0.01 [0.01, 0.01]	0.08 [0.01, 0.75]	0.01 [0.01, 0.01]
6'-SL				
Mean (SD)	0.26 (0.10)	0.25 (0.10)	0.30 (0.12)	0.27 (0.05)
Median [Min, Max]	0.24 [0.05, 0.70]	0.23 [0.07, 0.55]	0.28 [0.08, 0.73]	0.28 [0.19, 0.38]
LNT				
Mean (SD)	0.85 (0.41)	1.32 (0.593)	0.75 (0.45)	2.00 (0.40)
Median [Min, Max]	0.79 [0.09, 2.90]	1.20 [0.24, 3.10]	0.66 [0.20, 2.00]	1.90 [1.50, 2.70]
LNnT				
Mean (SD)	0.10 (0.06)	0.03 (0.02)	0.07 (0.04)	0.03 (0.03)
Median [Min, Max]	0.09 [0.01, 0.38]	0.02 [0.01, 0.16]	0.06 [0.02, 0.20]	0.02 [0.01, 0.08]
LNFP I				
Mean (SD)	0.55 (0.38)	0.04 (0)	1.40 (0.72)	0.04 (0)
Median [Min, Max]	0.45 [0.04, 2.30]	0.04 [0.04, 0.04]	1.30 [0.37, 4.10]	0.04 [0.04, 0.04]
LNFP V				

Mean (SD)	0.02 (0.01)	0.09 (0.04)	0.01 (0.01)	0.07 (0.06)
Median [Min, Max]	0.02 [0.01, 0.09]	0.09 [0.03, 0.24]	0.01 [0.01, 0.05]	0.06 [0.01, 0.21]
LNFP III				
Mean (SD)	0.18 (0.07)	0.21 (0.08)	0.15 (0.09)	0.22 (0.07)
Median [Min, Max]	0.17 [0.04, 0.48]	0.20 [0.08, 0.56]	0.12 [0.03, 0.47]	0.19 [0.11, 0.30]
LNFP II				
Mean (SD)	0.20 (0.16)	1.00 (0.40)	0.04 (0)	0.04 (0)
Median [Min, Max]	0.15 [0.04, 1.20]	0.95 [0.24, 2.50]	0.04 [0.04, 0.04]	0.04 [0.04, 0.04]
LNDFHI				
Mean (SD)	0.71 (0.28)	0.02 (0)	0.02 (0)	0.02 (0)
Median [Min, Max]	0.65 [0.07, 1.90]	0.02 [0.02, 0.02]	0.02 [0.02, 0.02]	0.02 [0.02, 0.02]
LNDFHII + LNnDFHII				
Mean (SD)	0.02 (0.02)	0.25 (0.14)	0.01 (0)	0.01 (0.00)
Median [Min, Max]	0.01 [0.01, 0.24]	0.23 [0.04, 0.73]	0.01 [0.01, 0.01]	0.01 [0.01, 0.02]
Sum of HMOs				
Mean (SD)	6.46 (1.11)	5.12 (1.06)	7.37 (1.59)	3.32 (0.60)
Median [Min, Max]	6.37 [3.58, 11.1]	4.99 [2.76, 8.40]	7.08 [4.43, 12.6]	3.18 [2.41, 4.14]

HMO, human milk oligosaccharides. 2’-FL, 2’-fucosyllactose; 3-FL, 3-fucosyllactose; 3’-SL, 3’-sialyllactose; 6'-GL, 6’-Galactooligosaccharide; DFL, 3,2’-difucosyllactose; 6’-SL, 6’-sialyllactose; LNT, lacto-N-tetrose; LNnT, lacto-N-neotetraose; LNFP I, Lacto-N-Fucopentaose I; LNFP V, Lacto-N-Fucopentaose V; LNFP III, Lacto-N-Fucopentaose III; LNFP II, Lacto-N-Fucopentaose II; LNDFH I, Lacto-N-difucohexaose I; LNDFH II, Lacto-N-difucohexaose II; LNnDFH II, Lacto-N-neodifucohexaose II

Table S6. Absolute concentrations of human milk oligosaccharides according to milk groups at 6 months.

HMOs	Group I (*n*=330)	Group II (*n*=80)	Group III (*n*=32)	Group IV (*n*=6)
Lactose				
Mean (SD)	70.2 (4.15)	70.7 (4.87)	71.0 (3.37)	73.5 (4.18)
Median [Min, Max]	70.0 [51.0, 81.0]	71.0 [46.0, 85.0]	70.5 [63.0, 76.0]	72.5 [69.0, 81.0]
2'-FL				
Mean (SD)	1.93 (0.74)	0.130 (0)	3.34 (0.92)	0.13 (0)
Median [Min, Max]	1.90 [0.13, 3.90]	0.13 [0.13, 0.13]	3.35 [0.54, 4.8]	0.13 [0.13, 0.13]
3'-FL				
Mean (SD)	1.06 (0.45)	2.30 (0.51)	0.17 (0.08)	0.37 (0.12)
Median [Min, Max]	0.98 [0.33, 3.20]	2.30 [1.10, 3.90]	0.17 [0.05, 0.41]	0.38 [0.16, 0.59]
3'-SL				
Mean (SD)	0.16 (0.06)	0.16 (0.04)	0.16 (0.05)	0.16 (0.03)
Median [Min, Max]	0.15 [0.05, 0.57]	0.15 [0.09, 0.23]	0.15 [0.08, 0.38]	0.15 [0.12, 0.21]
6'-GL				
Mean (SD)	0.01 (0.00)	0.01 (0.01)	0.01 (0.00)	0.01 (0.04)
Median [Min, Max]	0.01 [0.00, 0.04]	0.01 [0.00, 0.04]	0.01 [0.00, 0.02]	0.01 [0.01, 0.02]
DFL				
Mean (SD)	0.31 (0.22)	0.01 (0)	0.10 (0.13)	0.01 (0)
Median [Min, Max]	0.26 [0.02, 2.90]	0.01 [0.01, 0.01]	0.07 [0.01, 0.73]	0.01 [0.01, 0.01]
6'-SL				
Mean (SD)	0.03 (0.02)	0.03 (0.02)	0.04 (0.03)	0.05(0.02)
Median [Min, Max]	0.03 [0.01, 0.29]	0.03 [0.010, 0.11]	0.03 [0.01, 0.12]	0.04 [0.03, 0.08]
LNT				
Mean (SD)	0.42 (0.26)	0.56 (0.29)	0.50 (0.32)	1.35 (0.35)
Median [Min, Max]	0.38 [0.05, 1.60]	0.51 [0.12, 1.50]	0.46 [0.17, 1.40]	1.40 [0.71, 1.80]
LNnT				
Mean (SD)	0.05 (0.05)	0.016 (0.01)	0.05 (0.03)	0.03 (0.03)
Median [Min, Max]	0.04 [0.01, 0.24]	0.01 [0.01, 0.08]	0.03 [0.01, 0.12]	0.020 [0.01, 0.08]
LNFP I				
Mean (SD)	0.18 (0.18)	0.04 (0)	0.81 (0.35)	0.04 (0)

Median [Min, Max]	0.14 [0.04, 1.00]	0.04 [0.04, 0.04]	0.77 [0.21, 1.90]	0.04 [0.04, 0.04]
LNFP V				
Mean (SD)	0.02 (0.00)	0.05 (0.02)	0.01 (0.00)	0.04 (0.02)
Median [Min, Max]	0.01 [0.01, 0.06]	0.05 [0.01, 0.12]	0.01 [0.01, 0.02]	0.03 [0.01, 0.06]
LNFP III				
Mean (SD)	0.19 (0.07)	0.20 (0.06)	0.21 (0.08)	0.30 (0.11)
Median [Min, Max]	0.18 [0.06, 0.45]	0.19 [0.08, 0.34]	0.20 [0.03, 0.34]	0.32 [0.14, 0.43]
LNFP II				
Mean (SD)	0.17 (0.11)	0.61 (0.24)	0.04 (0)	0.04 (0)
Median [Min, Max]	0.14 [0.04, 0.73]	0.59 [0.19, 1.40]	0.04 [0.04, 0.04]	0.04 [0.04, 0.04]
LNDFHI				
Mean (SD)	0.40 (0.16)	0.02 (0)	0.02 (0)	0.02 (0)
Median [Min, Max]	0.38 [0.03, 0.99]	0.02 [0.02, 0.02]	0.02 [0.02, 0.02]	0.02 [0.02, 0.02]
LNDFHII + LNnDFHII				
Mean (SD)	0.03 (0.02)	0.19 (0.08)	0.01 (0.00)	0.01 (0.00)
Median [Min, Max]	0.03 [0.01, 0.19]	0.18 [0.06, 0.44]	0.01 [0.01, 0.01]	0.01 [0.01, 0.01]
Sum of HMOs				
Mean (SD)	4.98 (0.81)	4.32 (0.68)	5.51 (1.02)	2.55 (0.40)
Median [Min, Max]	4.94 [3.01, 8.35]	4.29 [2.91, 6.01]	5.46 [2.62, 7.74]	2.67 [1.84, 2.91]

HMO, human milk oligosaccharides. 2’-FL, 2’-fucosyllactose; 3-FL, 3-fucosyllactose; 3’-SL, 3’-sialyllactose; 6'-GL, 6’-Galactooligosaccharide; DFL, 3,2’-difucosyllactose; 6’-SL, 6’-sialyllactose; LNT, lacto-N-tetrose; LNnT, lacto-N-neotetraose; LNFP I, Lacto-N-Fucopentaose I; LNFP V, Lacto-N-Fucopentaose V; LNFP III, Lacto-N-Fucopentaose III; LNFP II, Lacto-N-Fucopentaose II; LNDFH I, Lacto-N-difucohexaose I; LNDFH II, Lacto-N-difucohexaose II; LNnDFH II, Lacto-N-neodifucohexaose II.

Table S7. Absolute concentrations of human milk oligosaccharides according to milk groups at 12 months

HMOs	Group I (*n*=55)	Group II (*n*=13)	Group III (*n*=5)
Lactose			
Mean (SD)	62.6 (10.2)	56.4 (18.6)	64.4 (2.19)
Median [Min, Max]	65.0 [16.0, 76.0]	65.0 [14.0, 73.0]	63.0 [63.0, 68.0]
2'-FL			
Mean (SD)	1.54 (0.63)	0.13 (0)	2.64 (0.29)
Median [Min, Max]	1.50 [0.13, 3.30]	0.13 [0.13, 0.13]	2.60 [2.40, 3.10]
3'-FL			
Mean (SD)	1.25 (0.643)	3.43 (1.57)	0.245 (0.12)
Median [Min, Max]	1.00 [0.52, 3.70]	3.10 [0.63, 6.90]	0.27 [0.12, 0.40]
3'-SL			
Mean (SD)	0.26 (0.12)	0.22 (0.12)	0.17 (0.06)
Median [Min, Max]	0.22 [0.10, 0.59]	0.18 [0.10, 0.53]	0.17 [0.09, 0.23]
6'-GL			
Mean (SD)	0.01 (0.01)	0.02 (0.01)	0.01 (0.00)
Median [Min, Max]	0.01 [0.00, 0.03]	0.01 [0.00, 0.03]	0.01 [0.01, 0.01]
DFL			
Mean (SD)	0.49 (0.40)	0.01 (0)	0.12 (0.07)
Median [Min, Max]	0.33 [0.10, 2.20]	0.01 [0.01, 0.01]	0.12 [0.05, 0.22]
6'-SL			
Mean (SD)	0.01 (0.00)	0.01 (0.03)	0.01 (0.00)
Median [Min, Max]	0.01 [0.01, 0.03]	0.01 [0.01, 0.02]	0.01 [0.01, 0.02]
LNT			
Mean (SD)	0.37 (0.24)	0.35 (0.27)	0.30 (0.16)
Median [Min, Max]	0.32 [0.05, 1.20]	0.34 [0.05, 0.87]	0.22 [0.13, 0.48]
LNnT			
Mean (SD)	0.03 (0.03)	0.01 (0.01)	0.02 (0.01)
Median [Min, Max]	0.02[0.01, 0.13]	0.01 [0.01, 0.01]	0.02 [0.01, 0.02]
LNFP I			
Mean (SD)	0.19 (0.17)	0.04 (0)	0.59 (0.31)

Median [Min, Max]	0.12 [0.04, 0.62]	0.04 [0.04, 0.04]	0.42 [0.28, 1.00]
LNFP V			
Mean (SD)	0.02 (0.01)	0.05 (0.02)	0.01 (0)
Median [Min, Max]	0.02 [0.01, 0.03]	0.05 [0.02, 0.08]	0.01 [0.01, 0.01]
LNFP III			
Mean (SD)	0.15 (0.06)	0.12 (0.04)	0.15 (0.04)
Median [Min, Max]	0.14 [0.04, 0.30]	0.12 [0.05, 0.19]	0.16 [0.09, 0.18]
LNFP II			
Mean (SD)	0.19 (0.11)	0.58 (0.32)	0.04 (0)
Median [Min, Max]	0.16 [0.04, 0.43]	0.51 [0.20, 1.20]	0.04 [0.04, 0.04]
LNDFHI			
Mean (SD)	0.46 (0.19)	0.02 (0)	0.02 (0)
Median [Min, Max]	0.420 [0.0620, 0.900]	0.02 [0.02, 0.02]	0.02 [0.02, 0.02]
LNDFHII + LNnDFHII			
Mean (SD)	0.04 (0.03)	0.29 (0.09)	0.01 (0)
Median [Min, Max]	0.03 [0.01, 0.21]	0.26 [0.17, 0.46]	0.01 [0.01, 0.01]
Sum of HMOs			
Mean (SD)	5.00 (0.83)	5.27 (1.61)	4.33 (0.76)
Median [Min, Max]	5.04 [3.16, 6.95]	5.13 [1.66, 8.64]	4.36 [3.64, 5.48]

HMO, human milk oligosaccharides. 2′-FL, 2′-fucosyllactose; 3-FL, 3-fucosyllactose; 3′-SL, 3′-sialyllactose; 6'-GL, 6′-Galactooligosaccharide; DFL, 3,2′-difucosyllactose; 6′-SL; 6′-sialyl-lactose; LNT, lacto-N-tetrose; LNnT, lacto-N-neotetraose; LNFP I, Lacto-N-Fucopentaose I; LNFP V, Lacto-N-Fucopentaose V; LNFP III, Lacto-N-Fucopentaose III; LNFP II, Lacto-N-Fucopentaose II; LNDFH I, Lacto-N-difucohexaose I; LNDFH II, Lacto-N-difucohexaose II; LNnDFH II, Lacto-N-neodifucohexaose II.

Table S8: Absolute concentrations of human milk oligosaccharides (HMOs) in human milk samples from the Ulm SPATZ Health Study by secretor status

HMOs	6 weeks		*p* value	6 months		*p* value	12 months		*p* value
	Non-secretor (*n*=131)	Secretor (*n*=551)		Non-secretor (*n*=86)	Secretor (*n*=362)		Non-secretor (*n*=13)	Secretor (*n*=60)	
Lactose									
Mean (SD)	67.2 (3.78)	66.3 (3.79)	0.022	70.9 (4.86)	70.3 (4.09)	0.145	56.4 (18.6)	62.8 (9.77)	0.474
Median [Min, Max]	67.0 [56.0, 79.0]	67.0 [52.0, 77.0]		71.0 [46.0, 85.0]	70.0 [51.0, 81.0]		65.0 [14.0, 73.0]	65.0 [16.0, 76.0]	
2'-FL									
Mean (SD)	0.130 (0)	2.83 (1.00)	<0.001	0.130 (0)	2.05 (0.86)	<0.001	0.13 (0)	1.63 (0.68)	<0.001
Median [Min, Max]	0.13 [0.13, 0.13]	2.70 [0.13, 6.60]		0.13 [0.13, 0.13]	2.00 [0.13, 4.80]		0.13 [0.13, 0.13]	1.50 [0.13, 3.30]	
3'-FL									
Mean (SD)	1.48 (0.57)	0.45 (0.27)	<0.001	2.17 (0.70)	0.98 (0.50)	<0.001	3.43 (1.57)	1.17 (0.68)	<0.001
Median [Min, Max]	1.50 [0.08, 3.00]	0.40 [0.03, 2.40]		2.30 [0.16, 3.90]	0.94 [0.05, 3.20]		3.10 [0.63, 6.90]	0.10 [0.12, 3.70]	
3'-SL									
Mean (SD)	0.17 (0.05)	0.15 (0.04)	<0.001	0.16 (0.03)	0.16 (0.06)	0.661	0.22 (0.12)	0.25 (0.12)	0.225
Median [Min, Max]	0.16 [0.07, 0.32]	0.14 [0.05, 0.54]		0.15 [0.09, 0.23]	0.15 [0.05, 0.57]		0.18 [0.10, 0.53]	0.22 [0.09, 0.58]	
6'-GL									
Mean (SD)	0.02 (0.02)	0.02 (0.01)	0.058	0.01 (0.01)	0.01 (0.00)	0.041	0.02 (0.01)	0.01 (0.01)	0.116
Median [Min, Max]	0.02 [0.01, 0.15]	0.02 [0.00, 0.09]		0.01 [0.00, 0.04]	0.01 [0.00, 0.04]		0.01 [0.00, 0.03]	0.01 [0.00, 0.03]	
DFL									
Mean (SD)	0.01 (0)	0.23 (0.17)	<0.001	0.01 (0)	0.29 (0.22)	<0.001	0.01 (0)	0.46 (0.39)	<0.001
Median [Min, Max]	0.01 [0.01, 0.01]	0.20 [0.01, 1.80]		0.01 [0.01, 0.01]	0.25 [0.01, 2.90]		0.01 [0.01, 0.01]	0.32 [0.05, 2.20]	
6'-SL									
Mean (SD)	0.25 (0.10)	0.26 (0.10)	0.267	0.03 (0.02)	0.04 (0.02)	0.645	0.01 (0.00)	0.01 (0.00)	0.758
Median [Min, Max]	0.24 [0.07, 0.55]	0.25 [0.05, 0.73]		0.03 [0.01, 0.11]	0.030 [0.01, 0.29]		0.01 [0.01, 0.02]	0.01 [0.01, 0.03]	
LNT									
Mean (SD)	1.37 (0.61)	0.84 (0.42)	<0.001	0.62 (0.35)	0.43 (0.26)	<0.001	0.35 (0.30)	0.36 (0.23)	0.746
Median [Min, Max]	1.30 [0.24, 3.10]	0.76 [0.09, 2.90]		0.53 [0.12, 1.80]	0.38 [0.05, 1.60]		0.34 [0.05, 0.87]	0.32 [0.05, 1.20]	
LNnT									
Mean (SD)	0.01 (0.02)	0.03 (0.06)	<0.001	0.02 (0.01)	0.05 (0.05)	<0.001	0.01 (0.01)	0.03 (0.02)	<0.001
Median [Min, Max]	0.02 [0.01, 0.16]	0.09 [0.01, 0.39]		0.01 [0.01, 0.08]	0.04 [0.01, 0.24]		0.01 [0.01, 0.01]	0.02 [0.01, 0.13]	
LNFP I									
Mean (SD)	0.04 (0)	0.62 (0.49)	<0.001	0.04 (0)	0.25 (0.26)	<0.001	0.04 (0)	0.22 (0.21)	<0.001
Median [Min, Max]	0.04 [0.04, 0.04]	0.51 [0.04, 4.10]		0.04 [0.04, 0.04]	0.15 [0.04, 1.90]		0.04 [0.04, 0.04]	0.14 [0.04, 1.00]	
LNFP V									
Mean (SD)	0.10 (0.04)	0.02 (0.01)	<0.001	0.05 (0.02)	0.02 (0.01)	<0.001	0.05 (0.02)	0.02 (0.01)	<0.001
Median [Min, Max]	0.09 [0.01, 0.24]	0.02 [0.01, 0.09]		0.05 [0.01, 0.12]	0.013 [0.01, 0.06]		0.05 [0.02, 0.08]	0.01 [0.01, 0.03]	
HMOs	6 weeks			6 months			12 months		

	Non-secretor (*n*=131)	Secretor (*n*=551)	*p* value	Non-secretor (*n*=86)	Secretor (*n*=362)	*p* value	Non-secretor (*n*=13)	Secretor (*n*=60)	*p* value
LNFP III									
Mean (SD)	0.21 (0.08)	0.17 (0.08)	<0.001	0.20 (0.07)	0.19 (0.07)	0.284	0.12 (0.05)	0.12 (0.06)	0.206
Median [Min, Max]	0.20 [0.08, 0.56]	0.17 [0.03, 0.48]		0.19 [0.08, 0.43]	0.19 [0.03, 0.45]		0.10 [0.05, 0.19]	0.14 [0.04, 0.30]	
LNFP II									
Mean (SD)	0.94 (0.46)	0.19 (0.16)	<0.001	0.57 (0.27)	0.18 (0.11)	<0.001	0.58 (0.32)	0.18 (0.11)	<0.001
Median [Min, Max]	0.92 [0.04, 2.50]	0.13 [0.04, 1.20]		0.55 [0.04, 1.40]	0.13 [0.04, 0.73]		0.51 [0.20, 1.20]	0.15 [0.04, 0.43]	
LNDFHI									
Mean (SD)	0.02 (0)	0.65 (0.33)	<0.001	0.02 (0)	0.36 (0.18)	<0.001	0.02 (0)	0.43 (0.22)	<0.001
Median [Min, Max]	0.02 [0.02, 0.02]	0.63 [0.02, 1.90]		0.02 [0.02, 0.02]	0.36 [0.02, 0.99]		0.02 [0.02, 0.02]	0.41 [0.02, 0.90]	
LNDFHII + LNnDFHII									
Mean (SD)	0.23 (0.15)	0.020 (0.02)	<0.001	0.18 (0.09)	0.03 (0.02)	<0.001	0.29 (0.09)	0.03 (0.03)	<0.001
Median [Min, Max]	0.21 [0.01, 0.73]	0.01 [0.01, 0.24]		0.18 [0.01, 0.44]	0.02 [0.01, 0.19]		0.26 [0.17, 0.46]	0.03 [0.01, 0.210]	
Total HMOs									
Mean (SD)	4.99 (1.13)	6.54 (1.19)	<0.001	4.20 (0.81)	5.02 (0.84)	<0.001	5.27 (1.61)	4.95 (0.84)	0.32
Median [Min, Max]	4.91 [2.41, 8.40]	6.44 [3.58, 12.6]		4.25 [1.84, 6.01]	4.96 [2.62, 8.35]		5.13 [1.66, 8.64]	5.01 [3.16, 6.95]	

p values derived from Wilcoxon rank sum test comparing Secretor vs. Non-Secretor human milk. Bonferroni-adjusted level of statistical significance is α=0.05/16=0.0031. HMO, human milk oligosaccharides. 2′-FL, 2′-fucosyllactose; 3-FL, 3-fucosyllactose; 3′-SL, 3′-sialyllactose; 6'-GL, 6′-Galactooligosaccharide; DFL, 3,2′-difucosyllactose; 6′-SL; 6′-sialyllactose; LNT, lacto-N-tetrose; LNnT, lacto-N-neotetraose; LNFP I, Lacto-N-Fucopentaose I; LNFP V, Lacto-N-Fucopentaose V; LNFP III, Lacto-N-Fucopentaose III; LNFP II, Lacto-N-Fucopentaose II; LNDFH I, Lacto-N-difucohexaose I; LNDFH II, Lacto-N-difucohexaose II; LNnDFH II, Lacto-N-neodifucohexaose II.

Table S9: Relative proportion (%) of human milk oligosaccharides (HMOs) in human milk samples from the Ulm SPATZ Health study by secretor status

HMOs	6 weeks		6 months		12 months	
	**Non-secretor (n=131)**	**Secretor (*n*=551)**	**Non-secretor (*n*=86)**	**Secretor (*n*=363)**	**Non-secretor (*n*=13)**	**Secretor (*n*=60)**
2'-FL						
Mean (SD)	2.75 (0.671)	42.8 (11.9)	3.24 (0.77)	40.0 (13.2)	2.84 (1.56)	33.0 (13.3)
Median [Min, Max]	2.65 [1.55, 5.40]	43.1 [2.71, 74.2]	3.06 [2.16, 7.08]	40.2 [2.85, 77.3]	2.53 [1.50, 7.84]	33.0 [2.35, 71.4]
3'-FL						
Mean (SD)	29.6 (10.3)	7.38 (5.30)	50.7 (13.6)	20.5 (11.7)	62.4 (13.7)	23.6 (12.6)
Median [Min, Max]	29.4 [3.45, 53.5]	6.39 [0.31, 45.4]	53.3 [8.72, 75.5]	18.8 [0.85, 73.3]	60.6 [38.0, 81.8]	22.0 [3.29, 67.0]
3'-SL						
Mean (SD)	3.61 (1.14)	2.31 (0.68)	3.83 (1.15)	3.29 (1.06)	4.08 (1.34)	5.01 (2.07)
Median [Min, Max]	3.45 [1.48, 8.32]	2.23 [0.84, 7.02]	3.60 [1.66, 8.72]	3.10 [0.91, 9.38]	3.70 [2.63, 7.51]	4.54 [2.00, 10.2]
6'-GL						
Mean (SD)	0.40 (0.28)	0.27 (0.12)	0.26 (0.16)	0.19 (0.08)	0.23 (0.11)	0.19 (0.078)
Median [Min, Max]	0.34 [0.12, 2.45]	0.25 [0.08, 0.91]	0.23 [0.07, 1.03]	0.17 [0.03, 0.67]	0.20 [0.13, 0.57]	0.18 [0.09, 0.42]
DFL						
Mean (SD)	0.21 (0.05)	3.46 (2.07)	0.25 (0.06)	5.64 (3.25)	0.22 (0.12)	9.23 (8.68)
Median [Min, Max]	0.20 [0.12, 0.42]	3.10 [0.12, 18.3]	0.24 [0.17, 0.55]	5.27 [0.38, 34.7]	0.20 [0.12, 0.60]	6.00 [1.29, 56.5]
6'-SL						
Mean (SD)	5.26 (2.38)	4.04 (1.60)	0.82 (0.57)	0.71 (0.47)	0.24 (0.13)	0.25 (0.09)
Median [Min, Max]	4.79 [1.35, 15.8]	3.78 [0.78, 16.1]	0.71 [0.19, 4.09]	0.59 [0.15, 4.58]	0.21 [0.14, 0.60]	0.22 [0.14, 0.52]
LNT						
Mean (SD)	28.1 (13.1)	13.0 (6.27)	15.8 (11.8)	8.74 (5.21)	7.35 (5.13)	7.51 (4.86)
Median [Min, Max]	25.1 [4.43, 65.2]	12.0 [1.33, 41.8]	12.4 [2.43, 61.8]	7.92 [0.81, 45.8]	8.86 [0.89, 14.3]	7.08 [0.729, 25.1]
LNnT						
Mean (SD)	0.57 (0.55)	1.52 (0.92)	0.45 (0.55)	1.05 (0.83)	0.22 (0.122)	0.56 (0.48)

Median [Min, Max]	0.39 [0.12, 3.24]	1.33 [0.16, 7.72]	0.29 [0.17, 4.47]	0.80 [0.15, 5.17]	0.20 [0.12, 0.60]	0.43 [0.14, 2.7]
LNFP I						
Mean (SD)	0.85 (0.21)	8.88 (5.58)	0.10 (0.24)	4.68 (4.33)	0.87 (0.48)	4.53 (4.13)
Median [Min, Max]	0.81 [0.48, 1.66]	8.08 [0.69, 32.4]	0.94 [0.67, 2.18]	3.12 [0.62, 26.6]	0.78 [0.46, 2.41]	2.78 [0.58, 18.6]
LNFP V						
Mean (SD)	1.82 (0.65)	0.34 (0.24)	1.11 (0.36)	0.31 (0.16)	0.10 (0.51)	0.34 (0.15)
Median [Min, Max]	1.72 [0.54, 5.23]	0.27 [0.09, 1.58]	1.07 [0.28, 2.16]	0.27 [0.13, 1.33]	1.15 [0.28, 1.69]	0.30 [0.14, 0.82]
LNFP III						
Mean (SD)	4.30 (1.57)	2.73 (1.22)	5.15 (2.77)	3.90 (1.36)	2.61 (1.41)	2.99 (1.28)
Median [Min, Max]	4.04 [1.71, 9.18]	2.56 [0.34, 7.63]	4.57 [2.02, 20.2]	3.77 [0.51, 8.99]	2.67 [0.58, 5.79]	2.96 [0.54, 6.31]

HMOs	6 weeks		6 months		12 months	
	Non-secretor (*n*=131)	Secretor (*n*=551)	Non-secretor (*n*=86)	Secretor *n*=363)	Non-secretor (*n*=13)	Secretor (*n*=60)
LNFP II						
Mean (SD)	17.7 (6.09)	3.03 (2.73)	12.8 (4.67)	3.24 (2.37)	11.6 (5.80)	3.63 (2.22)
Median [Min, Max]	18.6 [0.97, 29.8]	2.14 [0.32, 20.6]	13.3 [1.37, 23.3]	2.68 [0.52, 16.6]	13.1 [3.56, 19.8]	3.11 [0.73, 9.97]
LNDFHI						
Mean (SD)	0.423 (0.103)	9.93 (4.26)	0.498 (0.119)	7.08 (3.09)	0.437 (0.240)	8.41 (3.70)
Median [Min, Max]	0.407 [0.238, 0.831]	10.4 [0.158, 22.5]	0.471 [0.333, 1.09]	7.52 [0.258, 15.4]	0.390 [0.231, 1.21]	8.59 [0.37, 15.3]
LNDFHII + LNnDFHII						
Mean (SD)	4.32 (2.02)	0.321 (0.389)	4.07 (1.51)	0.589 (0.485)	5.88 (1.90)	0.69 (0.57)
Median [Min, Max]	4.51 [0.241, 9.95]	0.192 [0.0791, 4.30]	4.24 [0.344, 7.75]	0.477 [0.129, 4.16]	5.61 [3.26, 10.3]	0.54 [0.14, 3.80]

HMO, human milk oligosaccharides. 2′-FL, 2′-fucosyllactose; 3-FL, 3-fucosyllactose; 3′-SL, 3′-sialyllactose; 6'-GL, 6′-Galactooligosaccharide; DFL, 3,2′-difucosyllactose; 6′-SL; 6′-sialyllactose; LNT, lacto-N-tetrose; LNnT, lacto-N-neotetraose; LNFP I, Lacto-N-Fucopentaose I; LNFP V, Lacto-N-Fucopentaose V; LNFP III, Lacto-N-Fucopentaose III; LNFP II, Lacto-N-Fucopentaose II; LNDFH I, Lacto-N-difucohexaose I; LNDFH II, Lacto-N-difucohexaose II; LNnDFH II, Lacto-N-neodifucohexaose II.

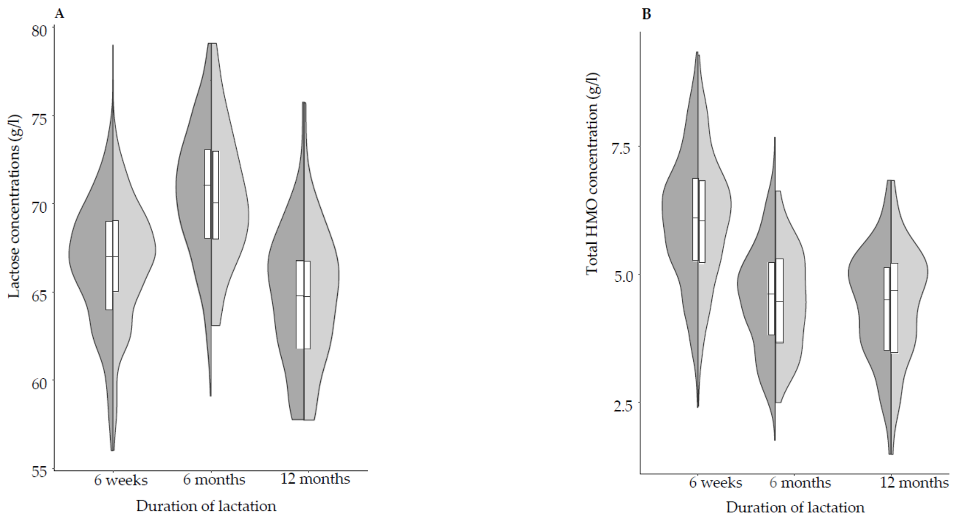

Figure S1. Split violin plots of the distribution of (a) lactose and (b) total human milk oligosaccharide concentrations (g/l) during the first 12 months of lactation regardless of milk type and secretor status. Shaded areas show the split violin plots of the density function with inserted boxed indicating the first and third quartile and the median. Dark grey ( ): Total sample size [6 weeks (*n* = 682), 6 months (*n* = 448), 12 months (*n* = 73)]; light grey ( ): Sub-sample size [6 weeks samples restricted to those who provided samples at 6 months (*n* = 422), 6 months samples restricted to those who provided samples at 12 months (*n* = 68), 12 months samples restricted to those who provided samples at 12 all three time point (*n* = 66)].

Table S10a: *p*-values from linear mixed effect models showing the effect of time, secretor status and milk group on absolute human milk oligosaccharide concentrations over 6 months of lactation among the (*n* = 422) lactating women

	Secretor	Time	Interaction	Milk Group	Time	Interaction
Lactose	0.0651	<.0001	0.7881	0.2865	< 0.001	0.1854
2'-FL	<.0001	0.9366	<.0001	< 0.001	< 0.001	< 0.001
3'-FL	<.0001	<.0001	<.0001	< 0.001	< 0.001	< 0.001
3'-SL	<.0001	0.0125	<.0001	< 0.001	< 0.001	< 0.001
6'-GL	0.1702	<.0001	0.3764	0.2414	< 0.001	0.8122
DFL	<.0001	< 0.001	<.0001	< 0.001	< 0.001	< 0.001
6'-SL	0.8712	<.0001	0.9850	0.0264	< 0.001	0.4473
LNT	<.0001	<.0001	0.0001	< 0.001	< 0.001	< 0.001
LNnT	<.0001	<.0001	0.0018	< 0.001	< 0.001	< 0.001
LNFP I	<.0001	< 0.001	<.0001	< 0.001	< 0.001	< 0.001
LNFP V	<.0001	<.0001	<.0001	< 0.001	< 0.001	< 0.001
LNFP III	<.0001	0.7814	0.0040	< 0.001	< 0.001	< 0.001
LNFP II	<.0001	<.0001	<.0001	< 0.001	0.0061	< 0.001
LNDFH I	<.0001	0.9607	<.0001	< 0.001	< 0.001	< 0.001
LNDFH II + LNnDFH II	<.0001	0.0064	<.0001	< 0.001	< 0.001	< 0.001
Total HMOs	<.0001	<.0001	<.0001	< 0.001	< 0.001	< 0.001

2′-FL, 2′-fucosyllactose; 3-FL, 3-fucosyllactose; 3′-SL, 3′-sialyllactose; 6'-GL, 6′-Galactooligosaccharide; DFL, 3,2′-difucosyllactose; 6′-SL; 6′-sialyllactose; LNT, lacto-N-tetrose; LNnT, lacto-N-neotetraose; LNFP I, Lacto-N-Fucopentaose I; LNFP V, Lacto-N-Fucopentaose V; LNFP III, Lacto-N-Fucopentaose III; LNFP II, Lacto-N-Fucopentaose II; LNDFH I, Lacto-N-difucohexaose I; LNDFH II, Lacto-N-difucohexaose II; LNnDFH II, Lacto-N-neodifucohexaose II. *p*-values represent mixed effects models where time and secretor status or milk group were considered fixed effects, while participant ID was considered a random effect. Blom transformations were applied to Lactose and individual human milk oligosaccharide structures before inclusion into the models. Bonferroni-adjusted level of statistical significance is α=0.05/16=0.0031.

Table S10b: *p*-values from linear mixed effect models showing the effect of time, secretor status and milk group on centered log-ratio (CLR) transformed human milk oligosaccharide concentrations over 6 months of lactation among the (*n* = 422) lactating women.

	**Secretor**	**Time**	**Interaction**	**Milk Group**	**Time**	**Interaction**
2'-FL	< 0.001	< 0.001	< 0.001	< 0.001	0.0071	< 0.001
3'-FL	< 0.001	< 0.001	< 0.001	< 0.001	< 0.001	< 0.001
3'-SL	< 0.001	< 0.001	< 0.001	< 0.001	< 0.001	< 0.001
6'-GL	< 0.001	< 0.001	0.2244	< 0.001	0.0272	< 0.001
DFL	< 0.001	< 0.001	< 0.001	< 0.001	< 0.001	< 0.001
6'-SL	< 0.001	< 0.001	0.9269	< 0.001	< 0.001	0.7451
LNT	< 0.001	< 0.001	0.0125	< 0.001	< 0.001	0.0006
LNnT	< 0.001	0.0558	< 0.001	< 0.001	< 0.001	0.0001
LNFP I	< 0.001	< 0.001	< 0.001	< 0.001	< 0.001	< 0.001
LNFP V	< 0.001	< 0.001	< 0.001	< 0.001	< 0.001	< 0.001
LNFP III	< 0.001	< 0.001	0.0006	< 0.001	< 0.001	< 0.001
LNFP II	< 0.001	0.0127	< 0.001	< 0.001	< 0.001	< 0.001
LNDFH I	< 0.001	< 0.001	< 0.001	< 0.001	< 0.001	< 0.001
LNDFH II + LNnDFH II	< 0.001	< 0.001	< 0.001	< 0.001	< 0.001	< 0.001

2′-FL, 2′-fucosyllactose; 3-FL, 3-fucosyllactose; 3′-SL, 3′-sialyllactose; 6'-GL, 6′-Galactooligosaccharide; DFL, 3,2′-difucosyllactose; 6′-SL; 6′-sialyllactose; LNT, lacto-N-tetrose; LNnT, lacto-N-neotetraose; LNFP I, Lacto-N-Fucopentaose I; LNFP V, Lacto-N-Fucopentaose V; LNFP III, Lacto-N-Fucopentaose III; LNFP II, Lacto-N-Fucopentaose II; LNDFH I, Lacto-N-difucohexaose I; LNDFH II, Lacto-N-difucohexaose II; LNnDFH II, Lacto-N-neodifucohexaose II. *p*-values represent mixed effects models where time and secretor status or milk group were considered fixed effects, while participant ID was considered a random effect. CLR transformations were applied relative human milk oligosaccharide concentrations before inclusion into the models. Bonferroni-adjusted level of statistical significance is α=0.05/14=0.0036.

Table S11a: *p*-values from linear mixed effect models showing the effect of time, secretor status and milk group on absolute human milk oligosaccharide concentrations over 12 months of lactation among the (*n* = 66) lactating women.

	**Secretor**	**Time**	**Interaction**	**Milk Group**	**Time**	**Interaction**
Lactose	0.1699	<.0001	0.1135	0.31810	<.0001	0.33010
2'-FL	<.0001	0.9964	<.0001	<.0001	<.0001	<.0001
3'-FL	<.0001	<.0001	0.0004	<.0001	0.0008	<.0001
3'-SL	0.7583	0.0002	0.5552	0.9152	<.0001	0.3980
6'-GL	0.9576	<.0001	0.5831	0.9984	<.0001	0.4672
DFL	<.0001	0.9853	0.0007	<.0001	<.0001	0.0025
6'-SL	0.5441	<.0001	0.5913	0.1170	<.0001	0.2044
LNT	0.0001	<.0001	0.0002	0.0001	<.0001	0.0002
LNnT	<.0001	<.0001	0.1559	0.1547	<.0001	<.0001
LNFP I	<.0001	0.9572	<.0001	<.0001	<.0001	<.0001
LNFP V	<.0001	0.0002	0.0026	<.0001	0.6579	0.0190
LNFP III	<.0001	<.0001	<.0001	<.0001	<.0001	<.0001
LNFP II	<.0001	0.0003	<.0001	<.0001	0.0520	0.0004
LNDFH I	<.0001	0.9878	<.0001	<.0001	<.0001	<.0001
LNDFH II + LNnDFH II	<.0001	0.0153	<.0001	<.0001	<.0001	<.0001
Total HMOs	<.0001	0.0008	0.0015	0.0188	<.0001	<.0001

2’-FL, 2’-fucosyllactose; 3-FL, 3-fucosyllactose; 3’-SL, 3’-sialyllactose; 6'-GL, 6’-Galactooligosaccharide; DFL, 3,2’-difucosyllactose; 6’-SL; 6’-sialyllactose; LNT, lacto-N-tetrose; LNnT, lacto-N-neo-tetraose; LNFP I, Lacto-N-Fucopentaose I; LNFP V, Lacto-N-Fucopentaose V; LNFP III, Lacto-N-Fucopentaose III; LNFP II, Lacto-N-Fucopentaose II; LNDFH I, Lacto-N-difucohexaose I; LNDFH II, Lacto-N-difucohexaose II; LNnDFH II, Lacto-N-neodifucohexaose II. *p*-values represent mixed effects models where time and secretor status or milk group were considered fixed effects, while participant ID was considered a random effect. Blom transformations were applied to Lactose and individual human milk oligosaccharide structures before inclusion into the models. Bonferroni-adjusted level of statistical significance is α=0.05/16=0.0031.

Table S11b: *p*-values from linear mixed effect models showing the effect of time, secretor status and milk group on centered log-ratio (CLR) transformed human milk oligosaccharide concentrations over 12 months of lactation among the (n = 66) lactating women.

	Secretor	Time	Interaction	Milk Group	Time	Interaction
2'-FL	<.0001	<.0001	<.0001	<.0001	<.0001	<.0001
3'-FL	<.0001	<.0001	0.0006	<.0001	<.0001	<.0001
3'-SL	0.0407	<.0001	0.7579	<.0001	<.0001	0.0629
6'-GL	0.1493	0.0124	0.0319	0.0006	0.0000	0.0352
DFL	<.0001	0.0067	0.0080	<.0001	<.0001	0.0262
6'-SL	0.1156	<.0001	0.7103	<.0001	<.0001	0.1131
LNT	<.0001	<.0001	0.0029	<.0001	<.0001	0.0072
LNnT	<.0001	0.1350	0.0002	<.0001	<.0001	0.0012
LNFP I	<.0001	0.0012	<.0001	<.0001	<.0001	<.0001
LNFP V	<.0001	0.0061	<.0001	<.0001	<.0001	<.0001
LNFP III	<.0001	0.0001	<.0001	<.0001	<.0001	<.0001
LNFP II	<.0001	0.2670	<.0001	<.0001	<.0001	<.0001
LNDFH I	<.0001	<.0001	<.0001	<.0001	<.0001	<.0001
LNDFH II + LNnDFH II	<.0001	<.0001	<.0001	<.0001	<.0001	<.0001

2′-FL, 2′-fucosyllactose; 3-FL, 3-fucosyllactose; 3′-SL, 3′-sialyllactose; 6'-GL, 6′-Galactooligosaccharide; DFL, 3,2′-difucosyllactose; 6′-SL; 6′-sialyllactose; LNT, lacto-N-tetrose; LNnT, lacto-N-neotetraose; LNFP I, Lacto-N-Fucopentaose I; LNFP V, Lacto-N-Fucopentaose V; LNFP III, Lacto-N-Fucopentaose III; LNFP II, Lacto-N-Fucopentaose II; LNDFH I, Lacto-N-difucohexaose I; LNDFH II, Lacto-N-difucohexaose II; LNnDFH II, Lacto-N-neodifucohexaose II. *p*-values represent mixed effects models where time and secretor status or milk group were considered fixed effects, while participant ID was considered a random effect. CLR transformation were applied to relative human milk oligosaccharide concentrations before inclusion into the models. Bonferroni-adjusted level of statistical significance is α=0.05/14=0.0036.

Table S12a: Effect of time and milk group within secretors, and effect of time within non-secretors, milk groups 1, 2 and 3 on absolute human milk oligosaccharide concentrations over 6 months of lactation among the (n = 422) lactating women

	Secretors				Non-secretors		Group 1		Group 2		Group 3	
	Time		Milk group	Interaction	Time		Time		Time		Time	
	β	pvalue	*p* value	*p* value	β	*p* value	β	*p* value	β	*p* value	β	*p* value
Lactose	0.721	< 0.001	0.250	0.133	0.88	< 0.001	0.89	< 0.001	0.82	< 0.001	1.22	< 0.001
2'-FL	-0.622	< 0.001	< 0.001	0.907			-0.63	< 0.001			-0.64	< 0.001
3'-FL	1.119	< 0.001	< 0.001	< 0.001	0.73	< 0.001	0.96	< 0.001	0.76	< 0.001	0.64	< 0.001
3'-SL	0.516	< 0.001	0.108	0.143	-0.30	0.02	0.37	< 0.001	-0.34	0.01	0.07	0.60
6'-GL	-1.160	< 0.001	0.952	0.742	-1.11	< 0.001	-1.18	< 0.001	-1.11	< 0.001	-1.23	< 0.001

DFL	0.657	< 0.001	0.056	0.006			0.45	< 0.001			0.04	0.64
6'-SL	-1.552	< 0.001	0.043	0.576	-1.59	< 0.001	-1.58	< 0.001	-1.62	< 0.001	-1.65	< 0.001
LNT	-1.314	< 0.001	0.001	< 0.001	-1.34	< 0.001	-1.01	< 0.001	-1.38	< 0.001	-0.41	0.0001
LNnT	-1.056	< 0.001	< 0.001	0.001	-0.52	< 0.001	-0.83	< 0.001	-0.54	< 0.001	-0.37	0.0003
LNFP I	-0.954	< 0.001	< 0.001	0.026			-0.84	< 0.001			-0.60	< 0.001
LNFP V	-0.440	< 0.001	< 0.001	0.092	-0.66	< 0.001	-0.34	< 0.001	-0.68	< 0.001	-0.14	0.07
LNFP III	-0.076	0.535	< 0.001	< 0.001	0.03	0.79	0.32	< 0.001	-0.05	0.65	1.12	< 0.001
LNFP II	-0.102	0.062	< 0.001	0.420	-0.47	< 0.001	-0.07	0.01	-0.51	< 0.001	0.00	0.0001
LNDFH I	-1.290	< 0.001	< 0.001	< 0.001			-0.86	< 0.001			0.00	< 0.001
LNDFH II + LNnDFH II	0.837	< 0.001	0.761	< 0.001	-0.16	0.0015	0.56	< 0.001	-0.18	0.0005	0.00	< 0.001
Total HMOs	-1.053	< 0.001	0.010	0.452	-0.65	< 0.001	-1.10	< 0.001	-0.64	< 0.001	-1.21	< 0.001

2’-FL, 2’-fucosyllactose; 3-FL, 3-fucosyllactose; 3’-SL, 3’-sialyllactose; 6'-GL, 6’-Galactooligosaccharide; DFL, 3,2’-difucosyllactose; 6’-SL; 6’-sialyllactose; LNT, lacto-N-tetrose; LNnT, lacto-N-neotetraose; LNFP I, Lacto-N-Fucopentaose I; LNFP V, Lacto-N-Fucopentaose V; LNFP III, Lacto-N-Fucopentaose III; LNFP II, Lacto-N-Fucopentaose II; LNDFH I, Lacto-N-difucohexaose I; LNDFH II, Lacto-N-difucohexaose II; LNnDFH II, Lacto-N-neodifucohexaose II. *p*-values represent mixed effects models where time and secretor status or milk group were considered fixed effects, while participant ID was considered a random effect. Blom transformations were applied to Lactose and individual human milk oligosaccharide structures before inclusion into the models.

Table S12b: Effect of time and milk group within secretors, and effect of time within non-secretors, milk groups 1, 2 and 3 on centered log-ratio (CLR) transformed human milk oligosaccharide concentrations over 6 months of lactation among the (*n* = 422) lactating women

	**Secretors**				**Non-secretors**		**Group 1**		**Group 2**		**Group 3**	
	Time		Milk group	Interaction	Time		Time		time		time	
	β	pvalue	*p* value	*p* value	β	*p* value	β	*p* value	β	*p* value	β	*p* value
2'-FL	-0.01	0.74	< 0.001	0.31	0.34	< 0.001	-0.03	0.02	0.36	< 0.001	-0.08	0.03
3'-FL	1.23	< 0.001	< 0.001	0.16	0.77	< 0.001	1.18	< 0.001	0.79	< 0.001	1.09	< 0.001
3'-SL	0.56	< 0.001	< 0.001	< 0.001	0.26	< 0.001	0.44	< 0.001	0.26	< 0.001	0.20	< 0.001
6'-GL	-0.20	< 0.001	< 0.001	0.01	-0.25	< 0.001	-0.29	< 0.001	-0.24	< 0.001	-0.46	< 0.001
DFL	0.80	< 0.001	0.01	0.00	0.34	< 0.001	0.65	< 0.001	0.36	< 0.001	0.34	0.00
6'-SL	-1.72	< 0.001	< 0.001	0.52	-1.74	< 0.001	-1.74	< 0.001	-1.76	< 0.001	-1.80	< 0.001
LNT	-0.54	< 0.001	0.51	0.00	-0.52	< 0.001	-0.40	< 0.001	-0.54	< 0.001	-0.11	0.12
LNnT	-0.54	< 0.001	0.98	0.04	-0.12	0.05	-0.42	< 0.001	-0.13	0.04	-0.20	0.03
LNFP I	-1.01	< 0.001	< 0.001	< 0.001	0.34	< 0.001	-0.76	< 0.001	0.36	< 0.001	-0.27	< 0.001
LNFP V	0.11	0.00	0.38	0.84	-0.28	< 0.001	0.10	< 0.001	-0.28	< 0.001	0.09	0.02
LNFP III	0.38	< 0.001	0.88	0.00	0.35	< 0.001	0.48	< 0.001	0.34	< 0.001	0.68	< 0.001
LNFP II	0.30	< 0.001	< 0.001	0.19	-0.09	0.00	0.26	< 0.001	-0.12	0.00	0.18	< 0.001
LNDFH I	-0.48	< 0.001	< 0.001	< 0.001	0.34	< 0.001	-0.26	< 0.001	0.36	< 0.001	0.18	< 0.001
LNDFH II + LNnDFH II	1.10	< 0.001	< 0.001	< 0.001	0.24	< 0.001	0.79	< 0.001	0.24	< 0.001	0.18	< 0.001

2′-FL, 2′-fucosyllactose; 3-FL, 3-fucosyllactose; 3′-SL, 3′-sialyllactose; 6'-GL, 6′-Galactooligosaccharide; DFL, 3,2′-difucosyllactose; 6′-SL; 6′-sialyllactose; LNT, lacto-N-tetrose; LNnT, lacto-N-neotetraose; LNFP I, Lacto-N-Fucopentaose I; LNFP V, Lacto-N-Fucopentaose V; LNFP III, Lacto-N-Fucopentaose III; LNFP II, Lacto-N-Fucopentaose II; LNDFH I, Lacto-N-difucohexaose I; LNDFH II, Lacto-N-difucohexaose II; LNnDFH II, Lacto-N-neodifucohexaose II. *p*-values represent mixed effects models where time and secretor status or milk group were considered fixed effects, while participant ID was considered a random effect. CLR transformation was applied to relative human milk oligosaccharide concentrations before inclusion into the models.

Table S13a: Effect of time and milk group within secretors, and effect of time within non-secretors, milk groups 1 and 2 on absolute human milk oligosaccharide concentrations over 12 months of lactation among the (n = 66) lactating women

	**Secretors**				**Non-secretors**		**Group 1**		**Group 2**	
	Time		Milk Group	Interaction	Time		Time		Time	
	β	pvalue			β	*p* value	β	*p* value	β	pvalue
Lactose	-0.13	0.54	0.57	0.64	-0.59	0.00	-0.20	0.04	-0.59	0.00
2'-FL	-0.42	< 0.001	< 0.001	0.25			-0.49	< 0.001		
3'-FL	0.56	< 0.001	< 0.001	0.61	0.44	< 0.001	0.53	< 0.001	0.44	< 0.001
3'-SL	0.86	< 0.001	0.70	0.14	0.52	< 0.001	0.68	< 0.001	0.52	0.00
6'-GL	-0.46	0.002	0.58	0.52	-0.41	0.03	-0.54	< 0.001	-0.41	0.03
DFL	0.49	< 0.001	0.05	0.30			0.41	< 0.001		
6'-SL	-0.91	< 0.001	0.01	0.02	-1.11	< 0.001	-1.03	< 0.001	-1.11	< 0.001
LNT	-0.72	< 0.001	0.08	0.15	-1.11	< 0.001	-0.58	< 0.001	-1.11	< 0.001
LNnT	-0.69	< 0.001	0.57	0.92	-0.48	< 0.001	-0.69	< 0.001	-0.48	< 0.001
LNFP I	-0.62	< 0.001	0.47	0.02			-0.44	< 0.001		
LNFP V	-0.03	0.78	0.03	0.90	-0.41	< 0.001	-0.02	0.70	-0.41	< 0.001
LNFP III	-0.44	0.01	0.06	0.04	-0.90	< 0.001	-0.17	0.03	-0.90	< 0.001
LNFP II	0.15	0.12	0.001	0.49	-0.35	< 0.001	0.10	0.03	-0.35	< 0.001
LNDFH I	-0.41	0.002	< 0.001	0.17			-0.27	< 0.001		
LNDFH II + LNnDFH II	0.56	< 0.001	0.48	0.002	0.05	0.46	0.37	< 0.001	0.05	0.46
Total HMOs	-0.32	0.06	0.14	0.14	0.02	0.90	-0.51	< 0.001	0.02	0.90

2′-FL, 2′-fucosyllactose; 3-FL, 3-fucosyllactose; 3′-SL, 3′-sialyllactose; 6'-GL, 6′-Galactooligosaccharide; DFL, 3,2′-difucosyllactose; 6′-SL; 6′-sialyllactose; LNT, lacto-N-tetrose; LNnT, lacto-N-neotetraose; LNFP I, Lacto-N-Fucopentaose I; LNFP V, Lacto-N-Fucopentaose V; LNFP III, Lacto-N-Fucopentaose III; LNFP II, Lacto-N-Fucopentaose II; LNDFH I, Lacto-N-difucohexaose I; LNDFH II, Lacto-N-difucohexaose II; LNnDFH II, Lacto-N-neodifucohexaose II. *p*-values represent mixed effects models where time and secretor status or milk group were considered fixed effects, while participant ID was considered a random effect. Blom transformations were applied to Lactose and individual human milk oligosaccharide structures before inclusion into the models.

Table S13b: Effect of time and milk group within secretors, and effect of time within non-secretors, milk groups 1 and 2 on centered log-ratio (CLR) transformed human milk oligosaccharide concentrations over 12 months of lactation among the (*n* = 66) lactating women

	Secretors				Non-secretors		Group 1		Group 2	
	Time		Milk Group	Interaction	Time		Time		Time	
	β	pvalue	pvalue	pvalue	β	pvalue	β	pvalue	β	pvalue
2'-FL	-0.11	0.01	< 0.001	0.78	0.25	< 0.001	-0.12	< 0.001	0.25	< 0.001
3'-FL	0.52	< 0.001	< 0.001	0.04	0.55	< 0.001	0.65	< 0.001	0.55	< 0.001
3'-SL	0.57	< 0.001	< 0.001	0.01	0.46	< 0.001	0.46	< 0.001	0.46	< 0.001
6'-GL	-0.01	0.84	0.001	0.08	0.05	0.53	-0.09	0.001	0.05	0.530
DFL	0.44	< 0.001	0.001	0.28	0.25	< 0.001	0.52	< 0.001	0.25	< 0.001
6'-SL	-1.18	< 0.001	< 0.001	0.05	-1.26	< 0.001	-1.30	< 0.001	-1.26	< 0.001
LNT	-0.37	< 0.001	0.924	0.15	-0.59	< 0.001	-0.28	< 0.001	-0.59	< 0.001
LNnT	-0.47	< 0.001	0.215	0.72	-0.14	0.03	-0.49	< 0.001	-0.14	0.029
LNFP I	-0.46	< 0.001	0.01	0.03	0.25	< 0.001	-0.32	< 0.001	0.25	< 0.001
LNFP V	0.15	0.002	0.35	0.72	-0.14	0.022	0.14	< 0.001	-0.14	0.022
LNFP III	-0.02	0.83	0.98	0.04	-0.14	0.013	0.10	0.004	-0.14	0.013
LNFP II	0.33	< 0.001	0.12	0.13	-0.10	0.077	0.26	< 0.001	-0.10	0.077
LNDFH I	-0.05	0.37	< 0.001	0.19	0.25	< 0.001	0.01	0.831	0.25	< 0.001
LNDFH II + LNnDFH II	0.66	< 0.001	0.06	< 0.001	0.31	< 0.001	0.48	< 0.001	0.31	< 0.001

2′-FL, 2′-fucosyllactose; 3-FL, 3-fucosyllactose; 3′-SL, 3′-sialyllactose; 6'-GL, 6′-Galactooligosaccharide; DFL, 3,2′-difucosyllactose; 6′-SL; 6′-sialyllactose; LNT, lacto-N-tetrose; LNnT, lacto-N-neotetraose; LNFP I, Lacto-N-Fucopentaose I; LNFP V, Lacto-N-Fucopentaose V; LNFP III, Lacto-N-Fucopentaose III; LNFP II, Lacto-N-Fucopentaose II; LNDFH I, Lacto-N-difucohexaose I; LNDFH II, Lacto-N-difucohexaose II; LNnDFH II, Lacto-N-neodifucohexaose II. *p*-values represent mixed effects models where time and secretor status or milk group were considered fixed effects, while participant ID was considered a random effect. CLR transformation was applied to relative human milk oligosaccharide concentrations before inclusion into the models.

Table S14. Associations of secretor status, parity, exclusive breastfeeding and infant sex with human milk oligosaccharide concentrations measured at 6 weeks in the Ulm SPATZ Health Study

HMO	Secretor Status		Parity		EBF		Infant sex	
	β^1	*p*	β^2	*p*	β^3	*p*	β^4	*p*
Lactose	0.23	0.02	0.13	0.09	-0.35	<.0001	0.08	0.32
2'-FL	-1.75	<.0001	-0.03	0.73	-0.002	0.98	-0.17	0.02
3'-FL	1.55	<.0001	0.05	0.55	-0.02	0.85	0.10	0.21
3'-SL	0.56	0.05	-0.01	0.91	0.14	0.12	-0.06	0.44
6'-GL	0.16	0.05	0.12	0.05	0.003	0.98	-0.02	0.76
DFL	-1.75	<.0001	-0.004	0.96	0.05	0.60	-0.01	0.86
6'-SL	-0.10	0.45	-0.10	0.22	0.10	0.26	-0.02	0.88
LNT	0.96	<.0001	0.08	0.29	0.14	0.11	0.07	0.38
LNnT	-1.49	<.0001	-0.09	0.27	0.01	0.94	-0.03	0.72
LNFP I	-1.67	<.0001	0.03	0.03	0.01	0.92	-0.14	0.06
LNFP V	1.68	<.0001	0.10	0.19	-0.05	0.54	0.10	0.18
LNFP III	0.48	<.0001	0.19	0.02	-0.03	0.73	-0.05	0.51
LNFP II	1.49	<.0001	0.07	0.37	-0.03	0.76	0.11	0.15
LNDFH I	-1.53	<.0001	0.02	0.81	-0.03	0.03	-0.04	0.61
LNDFH II + LNnDFH II	1.59	<.0001	0.11	0.11	0.08	0.36	0.08	0.26
Total HMOs	-1.21	<.0001	0.07	0.40	0.11	0.20	-0.21	0.02

EBF, Exclusive breastfeeding; HMO, human milk oligosaccharides; 2'-FL, 2'-fucosyllactose; 3-FL, 3-fucosyllactose; 3'-SL, 3'-sialyllactose; 6'-GL, 6'-Galactooligosaccharide; DFL, 3,2'-difucosyllactose; 6'-SL; 6'-sialyllactose; LNT, lacto-N-tetrose; LNnT, lacto-N-neotetraose; LNFP I, Lacto-N-Fucopentaose I; LNFP V, Lacto-N-Fucopentaose V; LNFP III, Lacto-N-Fucopentaose III; LNFP II, Lacto-N-Fucopentaose II; LNDFH I, Lacto-N-difucohexaose I; LNDFH II, Lacto-N-difucohexaose II; LNnDFH II, Lacto-N-neodifucohexaose II. *p* values derived from a general linear model comparing HMO concentrations in [1]non-secretor vs secretor milk, [2]milk of multiparous vs primiparous women, [3]milk of non-EBF vs EBF infants and [4]milk of boys vs girls. Bonferroni-adjusted level of statistical significance is α=0.05/16=0.0031.

Table S15. Unadjusted[1] and adjusted[2] effects of maternal characteristics on the trajectories of human milk oligosaccharides in milk group I and II over 6 months of lactation in the Ulm SPATZ Health Study

**GROUP I MILK (n = 306)**				
Characteristic	6 months			
	β[1]	*p* value	β[2]	*p* value
Pre-pregnancy BMI				
3′-SL	0.32	0.013	0.30	0.06
Parity				
LNDFH II + LNnDFHII	-0.18	0.013	-0.17	0.06
3′-FL	-0.18	0.0043	-0.15	0.03
2′-FL	0.148	0.04	0.10	0.28
Gestation				
Total HMOs	-0.36	0.02	-0.38	0.058
Maternal BMI at 6 weeks				
LNFP I	0.20	0.0096	0.21	0.028
3′-SL	0.41	0.0009	0.33	0.028
3′-FL	-0.13	0.02	-0.10	0.13
Maternal BMI at 6 months				
LNFP I	0.25	0.004	0.21	0.04
3′-SL	0.32	0.02	0.24	0.14
3′-FL	-0.16	0.02	-0.14	0.07
**GROUP II MILK (n = 72)**				
Pre-pregnancy BMI				
6′-GL	0.36	0.02	0.29	0.09
Parity				
6′-SL	0.33	0.02	0.10	0.57

HMO, human milk oligosaccharides; BMI, Body Mass Index; 2′-FL, 2′-fucosyllactose; 3-FL, 3-fucosyllactose; 3′-SL, 3′-sialyllactose; 6'-GL, 6′-Galactooligosaccharide; DFL, 3,2′-difucosyllactose; 6′-SL; 6′-sialyllactose; LNT, lacto-N-tetrose; LNnT, lacto-N-neotetraose; LNFP I, Lacto-N-Fucopentaose I; LNFP V, Lacto-N-Fucopentaose V; LNFP III, Lacto-N-Fucopentaose III; LNFP II, Lacto-N-Fucopentaose II; LNDFH I, Lacto-N-difucohexaose I; LNDFH II, Lacto-N-difucohexaose II; LNnDFH II, Lacto-N-neodifucohexaose II. A multi-level interaction test between maternal characteristics and time were used to evaluate HMO changes over lactation within milk group I and milk group II. Adjustments made for maternal age, gestation, infant age, infant sex, pre-pregnancy BMI. Bonferroni-adjusted level of statistical significance is α=0.05/16=0.0031

Table S16. Influence of maternal characteristics on the trajectories of human milk oligosaccharides in milk group I and II over 12 months of lactation in the Ulm SPATZ Health Study

Characteristic	6 months				12 months			
	β[1]	*p* value	β[2]	*p* value	β[1]	*p* value	β[2]	*p* value
**GROUP I MILK (*n* = 44)**								
Parity								
LNFP III	-0.8078	0.0053	-0.2164	0.4571	-0.8385	0.0037	-0.3607	0.2166
LNFP V	-0.7269	0.0132	-0.3487	0.2848	-0.7109	0.0147	-0.5768	0.0787
LNFP I	-0.3299	0.0548	-0.2715	0.1642	-0.5581	0.0013	-0.5581	0.0076
LNnT	-0.4715	0.0192	-0.4086	0.072	-0.5617	0.0053	-0.531	0.0203
LNT	-0.396	0.065	-0.3747	0.1339	-0.6105	0.0047	-0.6983	0.0069
Gestation								
LNDFH I	0.7196	0.0874	0.5774	0.1647	1.0422	0.0214	0.8879	0.0468
DFL	0.4572	0.4258	0.2924	0.3281	1.91941	0.0113	1.252	0.0085
2′-FL	0.3002	0.3178	0.2684	0.3446	0.7457	0.0224	0.7502	0.0291
**GROUP II MILK (*n* = 11)**								
Parity								
LNnT	0.8824	0.0576	1.4203	0.0859	1.0066	0.0337	1.4236	0.0853
Delivery mode								
6′-SL	-0.05109	0.8875	0.4712	0.4129	-0.0754	0.0481	0.0926	0.8676
Gestation								
6′-SL	-0.5633	0.2354	-0.2026	0.4612	-1.2867	0.0115	-0.1352	0.7861
6′-GL	-0.2562	0.7911	0.3356	0.9949	-2.7575	0.0095	-1.2541	0.4769
3′-SL	0.4577	0.5797	3280	0.703	-1.8837	0.0318	-0.6219	0.1102
Maternal BMI at 12 months								
6′-GL	0.4497	0.406	-0.5731	0.4426	-1.4358	0.0142	0.6956	0.3582

BMI, Body Mass Index; 2′-FL, 2′-fucosyllactose; 3-FL, 3-fucosyllactose; 3′-SL, 3′-sialyllactose; 6'-GL, 6′-Galactooligosaccharide; DFL, 3,2′-difucosyllactose; 6′-SL; 6′-sialyllactose; LNT, lacto-N-tetrose; LNnT, lacto-N-neotetraose; LNFP I, Lacto-N-Fucopentaose I; LNFP V, Lacto-N-Fucopentaose V; LNFP III, Lacto-N-Fucopentaose III; LNFP II, Lacto-N-Fucopentaose II; LNDFH I, Lacto-N-difucohexaose I; LNDFH II, Lacto-N-difucohexaose II; LNnDFH II, Lacto-N-neodifucohexaose II. A multi-level interaction test between maternal characteristics and time were used to evaluate HMO changes over lactation within milk group I and milk group II. Adjustments made for maternal age, gestation, parity, infant age, infant sex, maternal BMI. Bonferroni-adjusted level of statistical significance is α=0.05/16=0.0031.
